# Supplementary figures and images for: Collective peroxide detoxification determines microbial mutation rate plasticity in E. coli
Source: PLoS Biol. 2024 Jul 15;22(7):e3002711. doi: 10.1371/journal.pbio.3002711 (PMC11272383; doi:10.1371/journal.pbio.3002711)

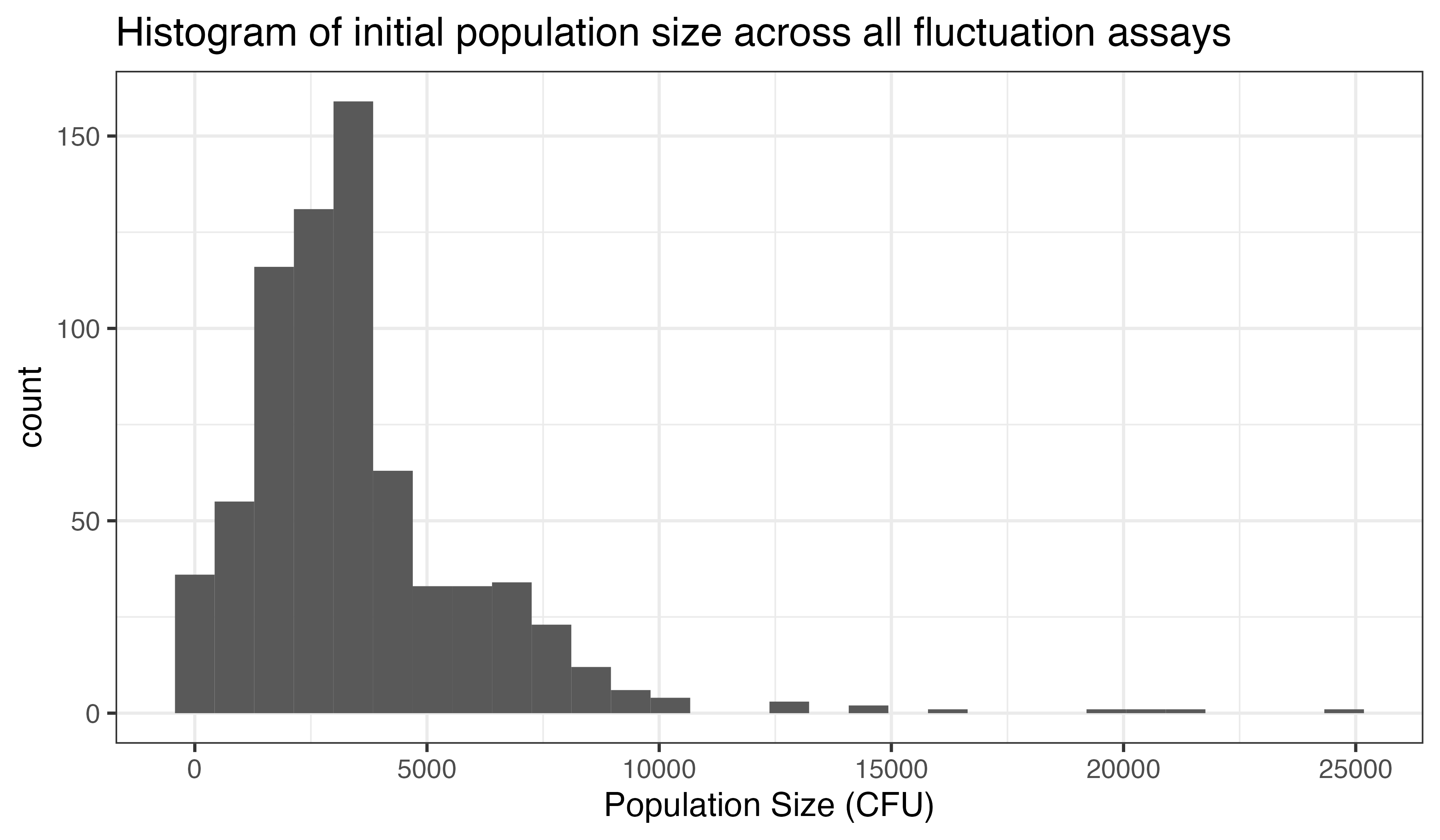

Supplement: S1 Fig — Mean = 3,537, median = 3,000. Low population size is desirable in order to maximise the number of generations considered and to reduce the chances of resistant mutants being present in the starting population (“jackpot cultures”). Raw data can be found in S5 Data. (TIFF) [file pbio.3002711.s001.tiff]

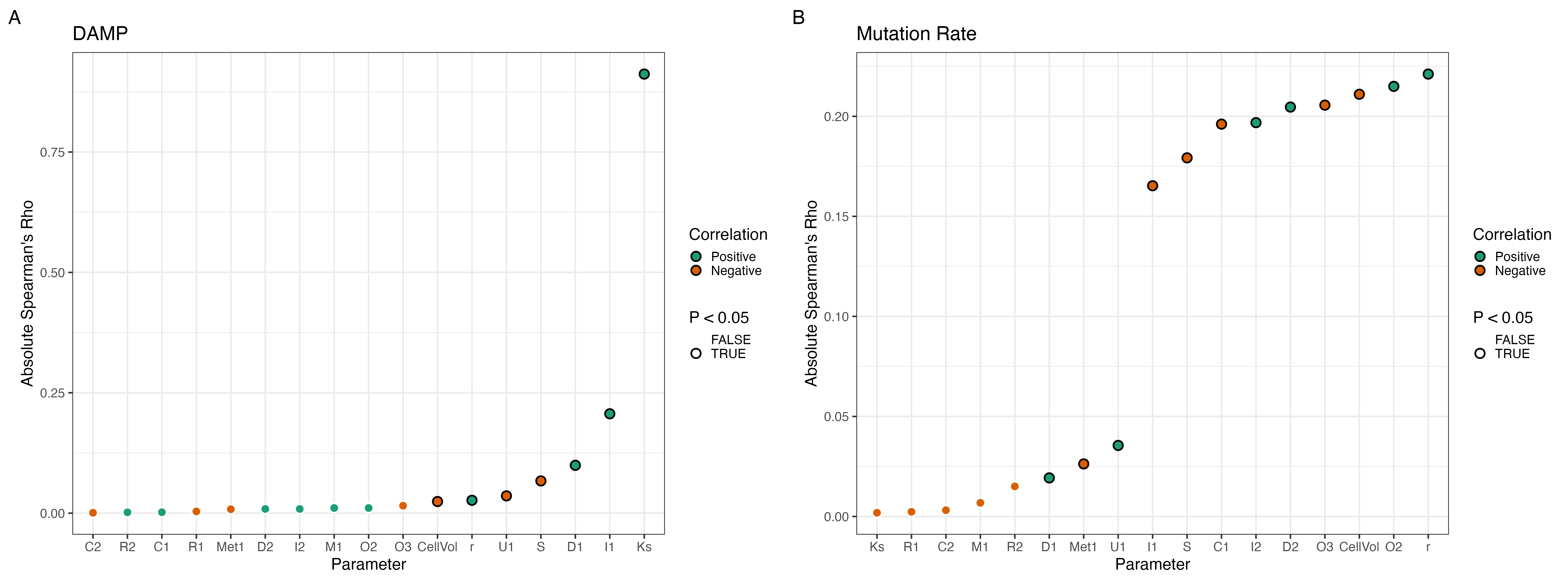

Supplement: S2 Fig — Left-hand side (A) shows the absolute rank correlation, as quantified by Spearman’s Rank Correlation Coefficient, between each parameter and the slope of DAMP, parameters are ordered from least to most correlation from left to right. Right-hand side (B) shows the equivalent information for the correlation between parameter values and mutation rate (at 250 mg L-1). Positive correlations are shown in green while negatively correlated parameters are shown in orange. Black borders show significant rank correlation (P < 0.05). Note the different y axis limits and x axis order on the left- VS right-hand side. Raw data can be found in S3 data, Spearman’s rank correlation coefficient statistics, and associated p-values can be found in S6 Data. (TIFF) [file pbio.3002711.s002.tiff]

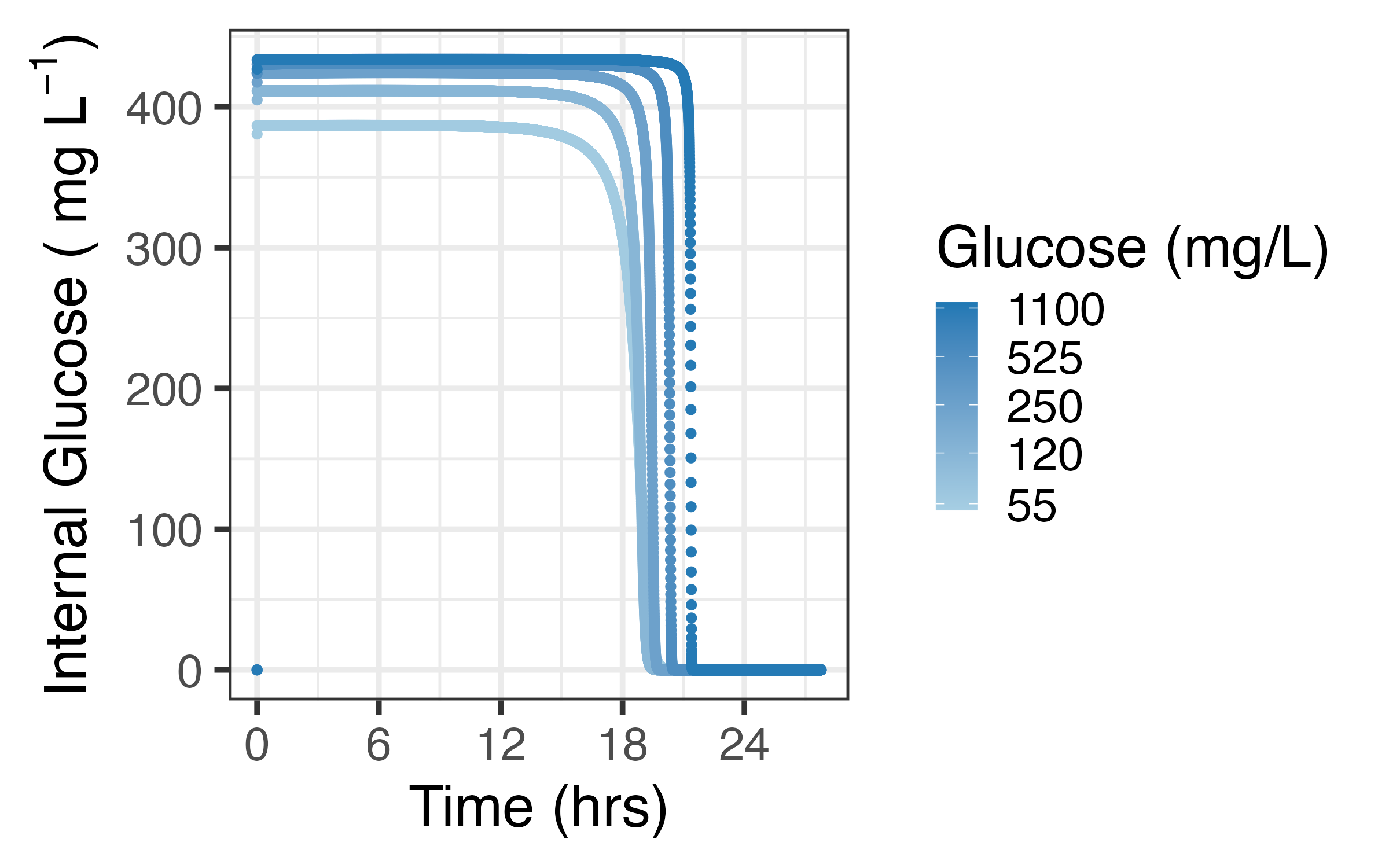

Supplement: S3 Fig — Higher levels of initial external glucose provision (point colour) lead to higher levels of internal glucose (y-axis). Raw data can be found in S1 Data. (TIFF) [file pbio.3002711.s003.tiff]

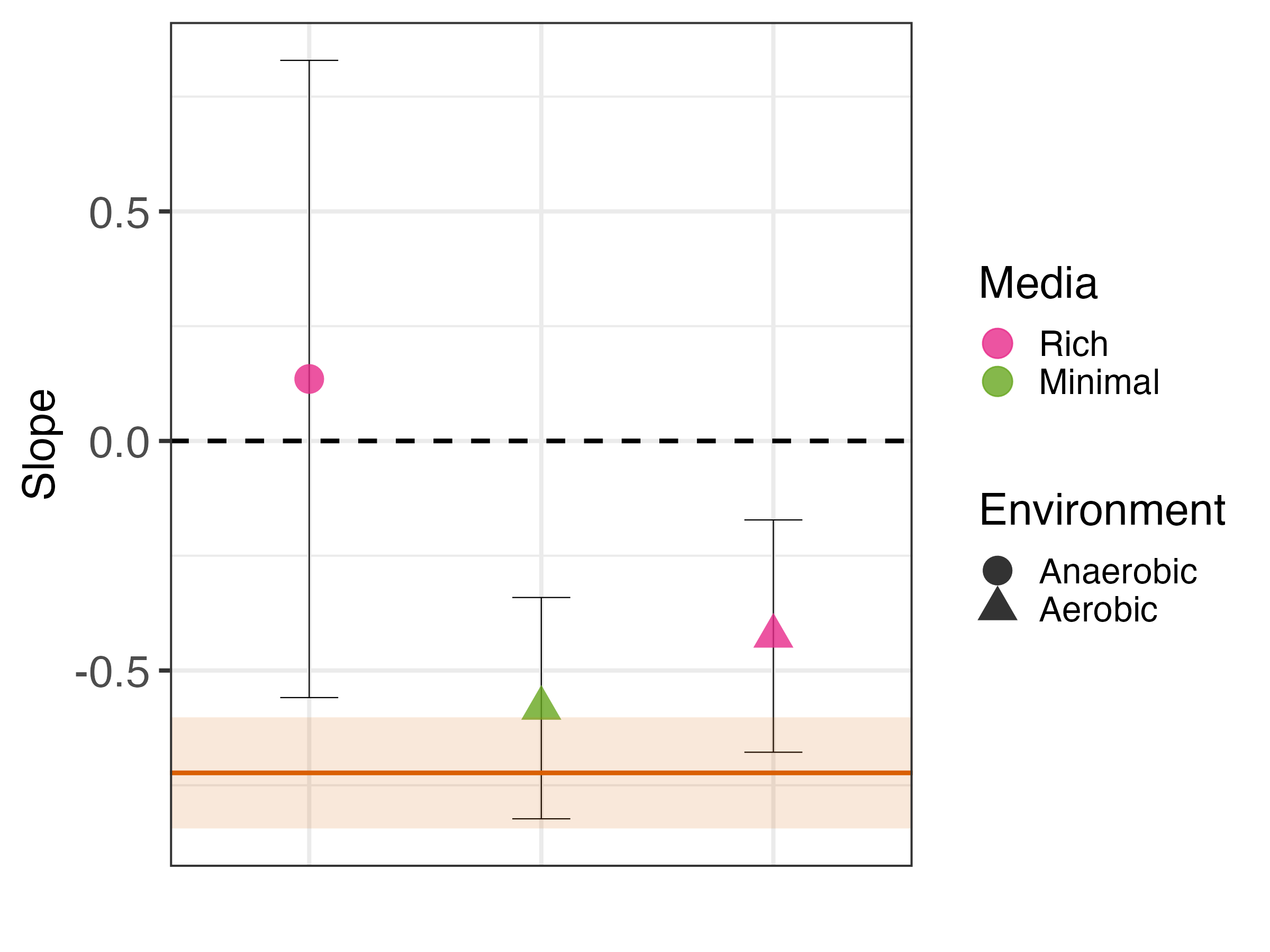

Supplement: S4 Fig — Pink circle = MG1655 rich media anaerobic (173 pc, 11 fa); green triangle = MG1655 minimal media aerobic (273 pc, 17 fa); pink triangle = MG1655 rich media aerobic (285 pc, 18 fa). Orange line and shaded area shows DAMP for BW25113 in rich media as in Fig 3 with 95% CI. Raw data can be found in S5 Data and summary statistics as plotted are in S1 Table. (TIFF) [file pbio.3002711.s004.tiff]

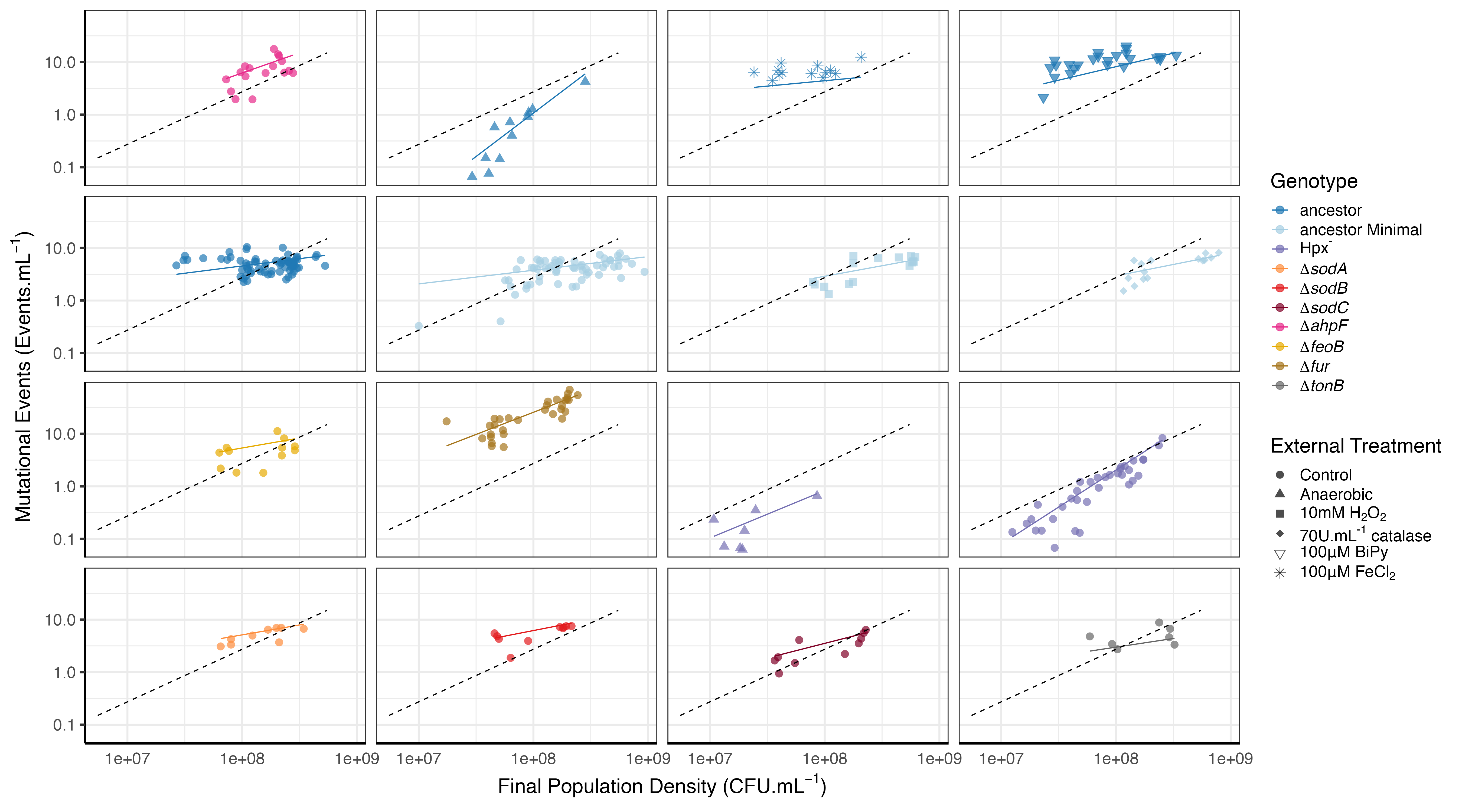

Supplement: S5 Fig — Final population density is plotted against mutational events per ml on a log-log scale. Dashed lines show the null expectation of a constant mutation rate (i.e., slope = 1), the y intercept for the dashed lines is arbitrary. Coloured lines are fitted slopes from mod3 (S1 Supplementary Statistics file), line gradients with 95% CI shown in Fig 3. Treatments shown are BW25113 ancestor (1122 parallel cultures (pc) across 70 fluctuation assays (fa)); ancestor minimal media (974 pc, 61 fa); ΔahpF (266 pc, 17 fa); Hpx- (546 pc, 35 fa); ancestor anaerobic (168 pc, 11 fa); ancestor 10 mM H2O2 (243 pc, 16 fa); ancestor 70U ml-1 catalase (231 pc, 15 fa); Hpx- anaerobic (105 pc, 7 fa); ancestor + chelator 2,2,Bipyridyl 100 μm (382 pc, 24 fa); ancestor + FeCl2 100 μm (210 pc, 13 fa); ΔfeoB (210 pc, 13 fa); Δfur (504 pc, 31 fa); ΔtonB (113 pc, 7 fa). Raw data can be found in S5 Data and summary statistics as plotted are in S1 Table. (TIFF) [file pbio.3002711.s005.tiff]

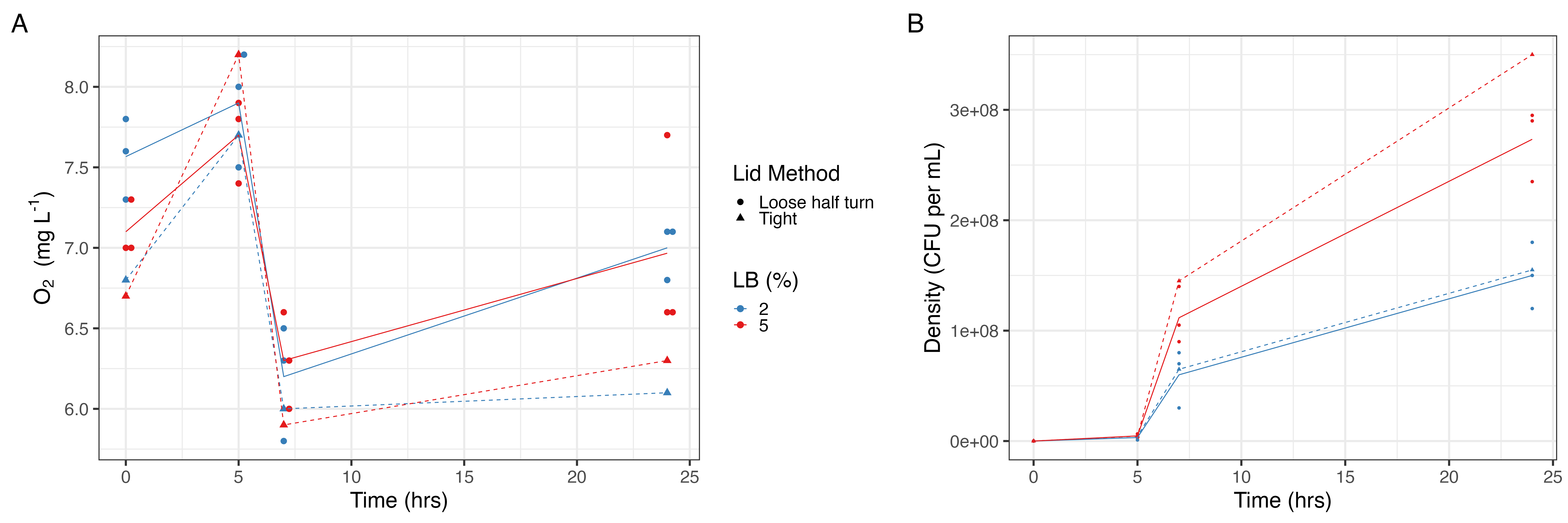

Supplement: S6 Fig — (A) O2 concentration is plotted as a function of time in wild-type BW25113 cultures grown in 2% LB (low density–blue points and lines) or 5% LB (high density–red points and lines); 50-ml tubes contain 10 ml of culture with lids attached by a small piece of tape and either screwed on tight (triangles) or loosened one half turn (circles). Solid lines connect mean values for half turn loosened samples and dashed lines connect points for tight samples. While there is consistent variation in oxygen concentration over time, there is no consistent variation among nutrient treatments. (B) Density measured at each time point alongside dissolved oxygen measurement. Raw data can be found in S7 Data. (TIFF) [file pbio.3002711.s006.tiff]

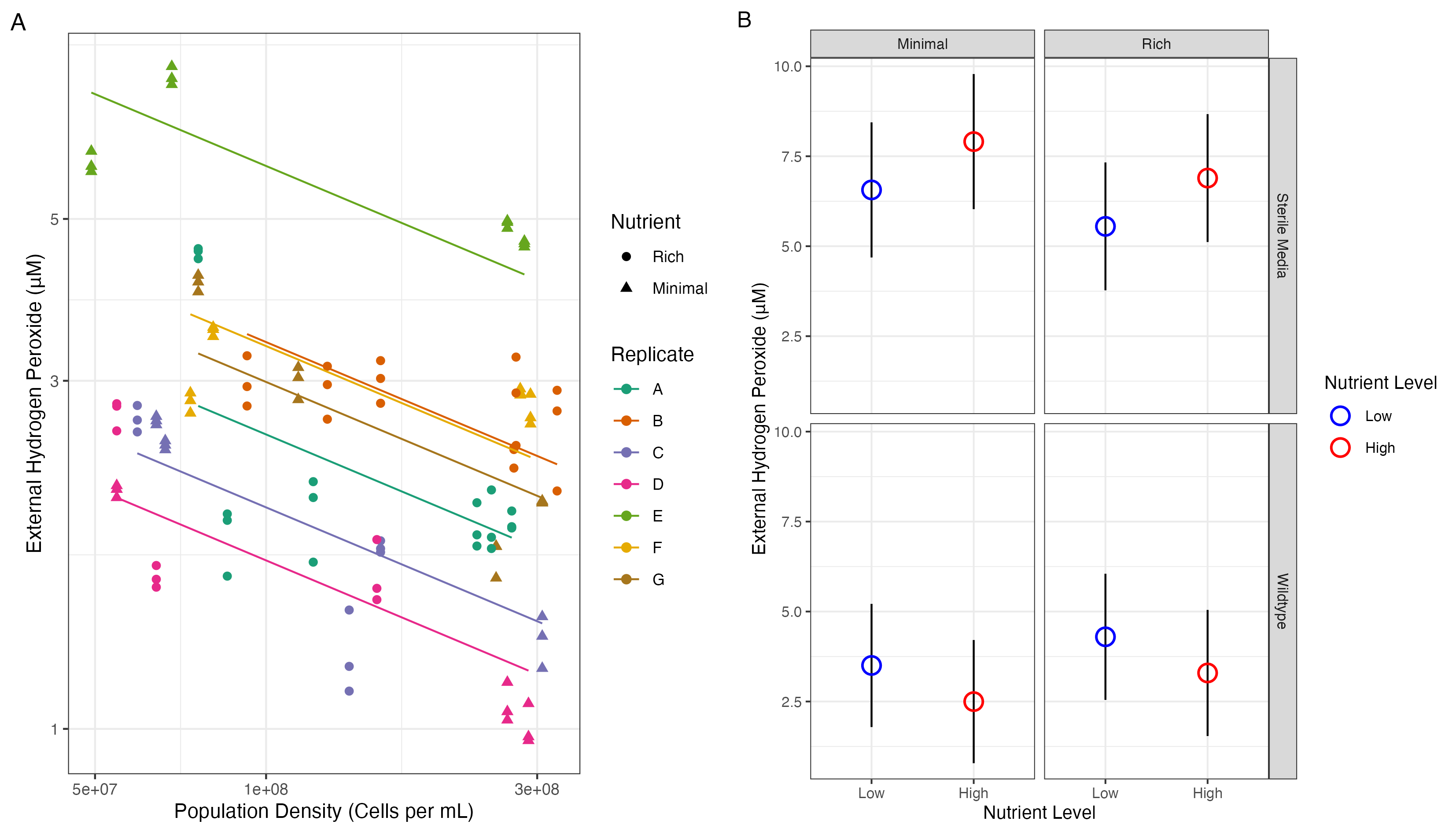

Supplement: S7 Fig — Left-hand side (A) shows the log-log relationship between population density and external H2O2 in cultures of MG1655 after 24 h of incubation. Rich media is 2/5% LB diluted in DM, minimal media is 80/1,000 mg L-1 glucose in DM. Population density is estimated from the optical density (OD 1 = 2.37 × 10^9^ cells ml-1 calculated from OD measurements taken alongside fluctuation assays). Lines of best fit are from regression 7B (SI). Right-hand side (B) shows the H2O2 concentration after 24 h incubation in rich or minimal media; sterile or with wild-type MG1655, Regression 6 (SI); error bars show 95% CI. The interaction effect between nutrient level (low versus high) and presence of a culture (Sterile Media versus Wild-type), where external peroxide decreases with nutrients increases in the presence of a culture but increases without one, is significant (F DF=46 = 9.8, P = 3 × 10^-3^, Regression 6 (SI)). Raw data can be found in S8 Data. (TIFF) [file pbio.3002711.s007.tiff]

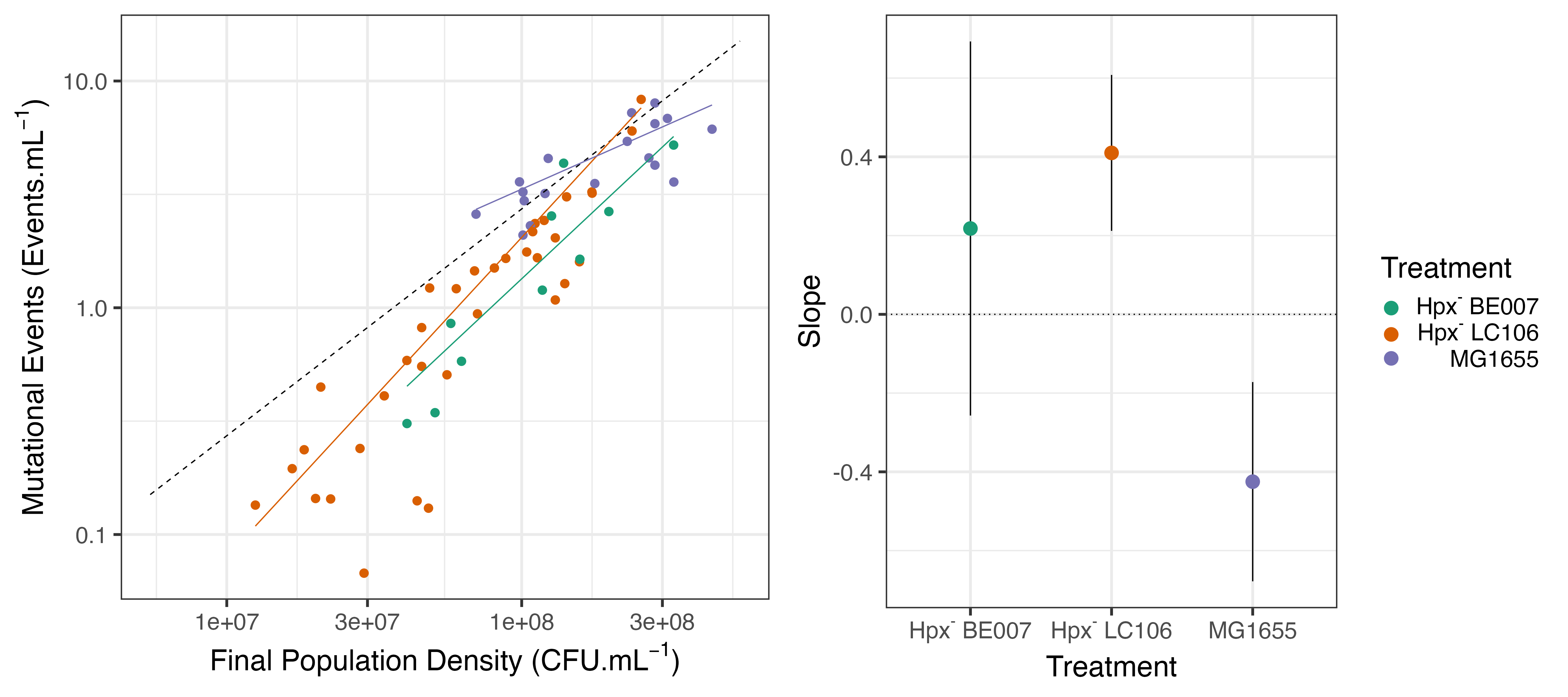

Supplement: S8 Fig — Left-hand plot shows raw data used to calculate DAMP slope in Hpx- LC106 (546 pc, 35 fa), Hpx- BE007 (149 pc, 10 fa), and their ancestor MG1655 (285 pc, 18 fa). Right hand shows DAMP slope as fitted by regression 4. DAMP slope does not significantly differ between the 2 Hpx- strains (LR = 0.71, DF = 110, P = 0.4); however, DAMP slopes do differ between MG1655 vs. LC106 (LR = 28.3, DF = 110, P < 0.0001) and MG1655 vs. BE007 (LR = 5.2, DF = 110, P = 0.02). Raw data can be found in S5 Data and summary statistics as plotted are in S1 Table. (TIFF) [file pbio.3002711.s008.tiff]

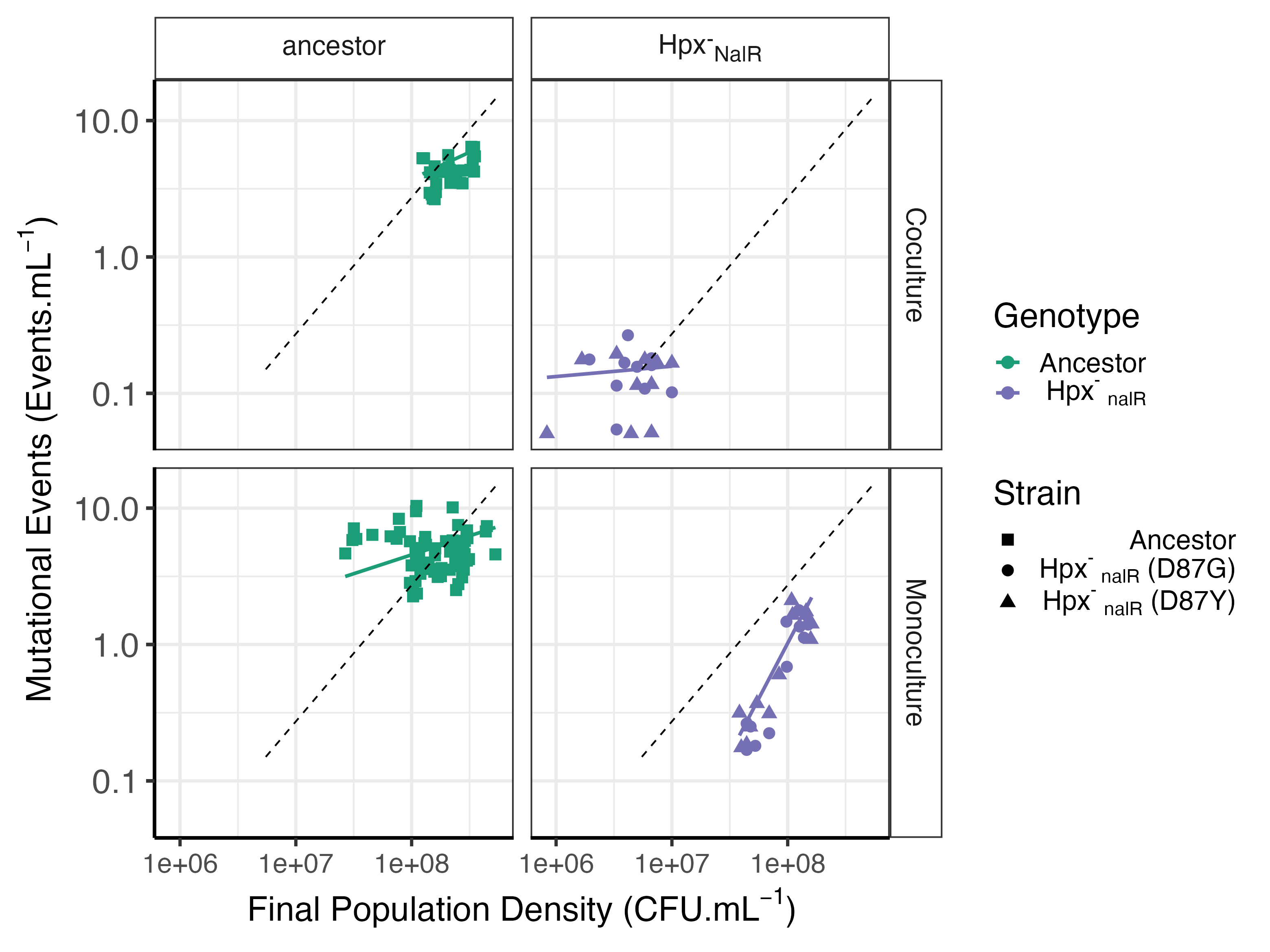

Supplement: S9 Fig — Final population density of the focal strain is plotted against mutational events per ml on a log-log scale. Dashed lines show the null expectation of a constant mutation rate with a slope of 1. Ancestor coculture measurements are taken in coculture with Hpx-, Hpx- D87Y, and D87G are cocultured with ancestor BW25113. Lines are fitted slopes shown in Fig 4. BW25113 ancestor (1,106 pc, 69 fa); BW25113 in coculture with Hpx- (498 pc, 31 fa); Hpx-nalR (388 pc, 24 fa); Hpx-nalR in coculture with BW25113 (319 pc, 20 fa). Raw data can be found in S5 Data and summary statistics as plotted are in S1 Table. (TIFF) [file pbio.3002711.s009.tiff]

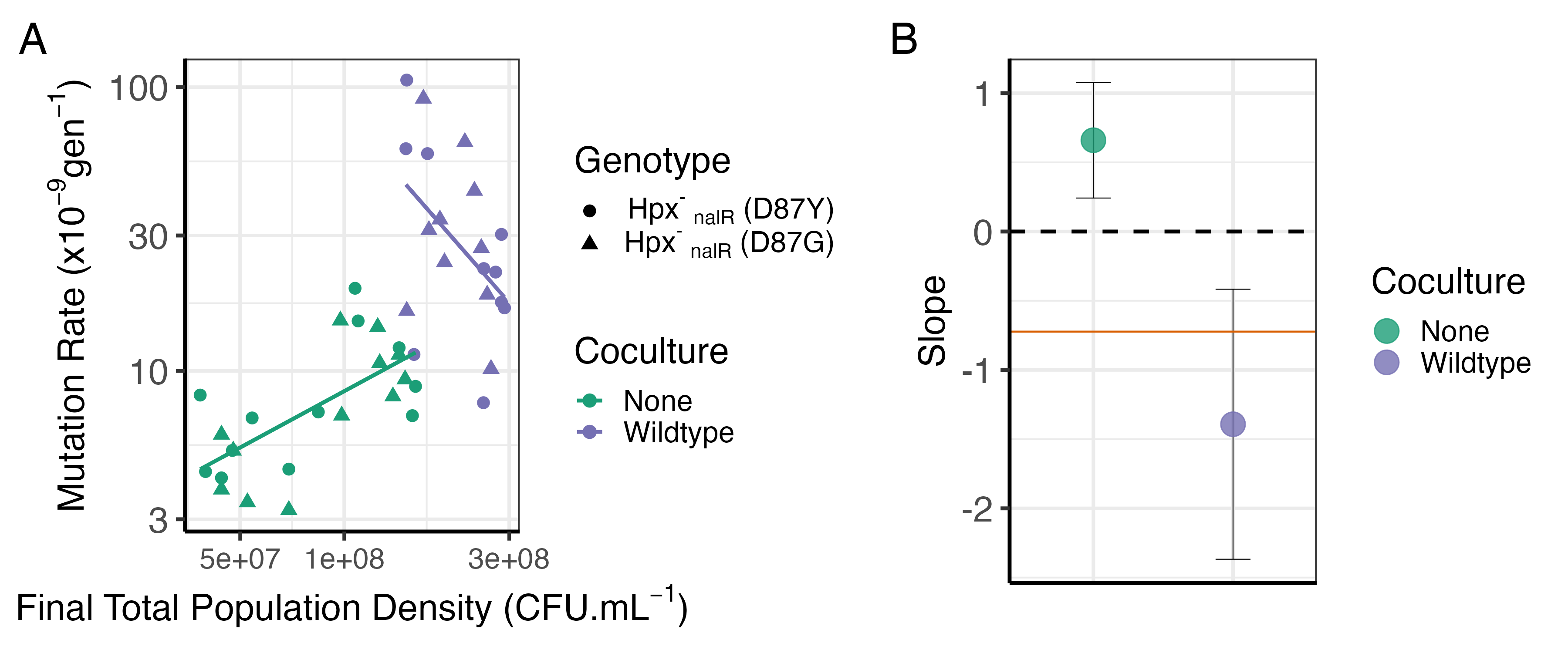

Supplement: S10 Fig — (A) Final population density (focal + coculture strain where relevant) is plotted against mutation rate on a log-log scale. Hpx-nalR monoculture (388 pc, 24 fa); Hpx-nalR in coculture with BW25113 (319 pc, 20 fa). Lines are fitted slopes shown from Regression 8 (SI). (B) Slope and 95% CI on the lines shown in LHS graph. Horizontal orange line shows the slope of the BW25113 ancestor in rich media (Regression 4 (SI), Fig 3). In monoculture Hpx- mutation rates increase with total population density while in coculture the wild type restores a negative association between density and mutation rates (DAMP). Raw data can be found in S5 Data. (TIFF) [file pbio.3002711.s010.tiff]

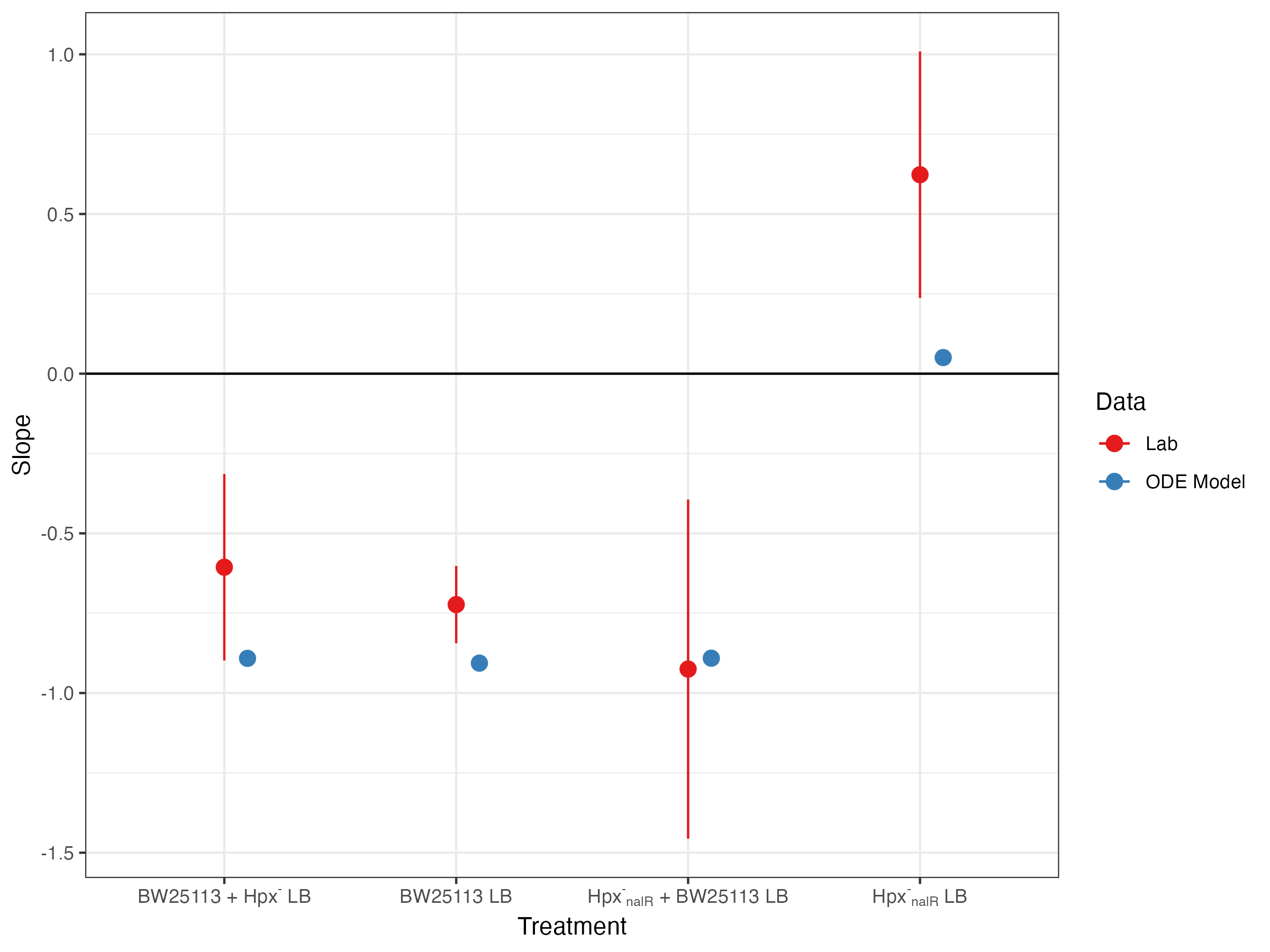

Supplement: S11 Fig — Estimates from lab data are shown in red as in Fig 4. Estimates from ODE modelling are shown in blue, and 95% CI are included for all points; however, ODE model CI are too narrow to be visible. Lab data summary statistics shown can be found in S1 Table and raw data in S5 Data; ODE model summary statistics shown can be found in S9 Data, raw data from ODE models can be found in S10 Data. (TIFF) [file pbio.3002711.s011.tiff]

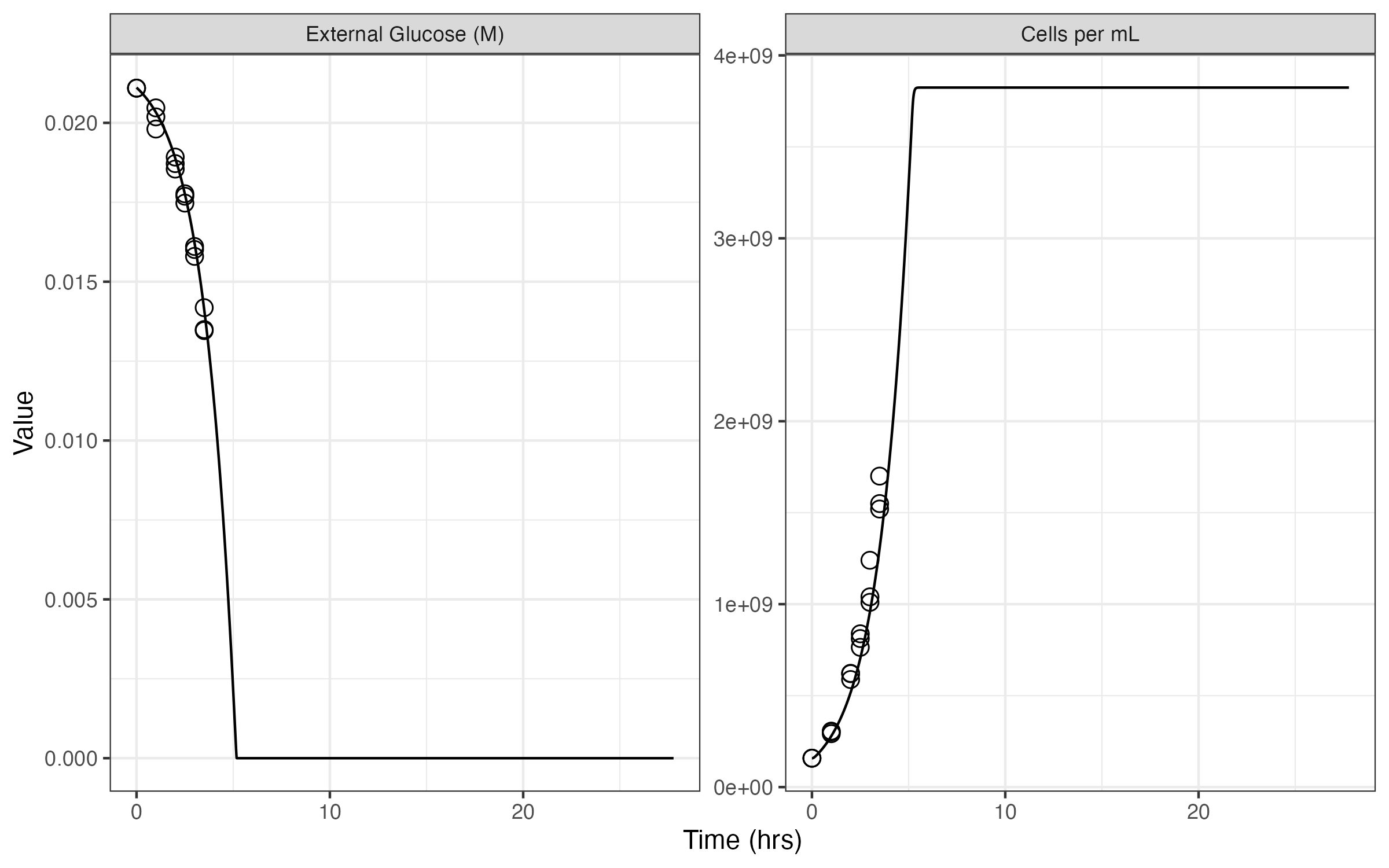

Supplement: S13 Fig — Lines show results of ODE model A simulated as described in methods, circles show data from [93] used to fit parameters U1 and M1. Left-hand panel shows the molar concentration of external glucose over time and right-hand panel shows E. coli cells per ml over time. Raw data for fitting can be found in S12 Data, data from ODE output can be found in S1 Data. (TIFF) [file pbio.3002711.s013.tiff]

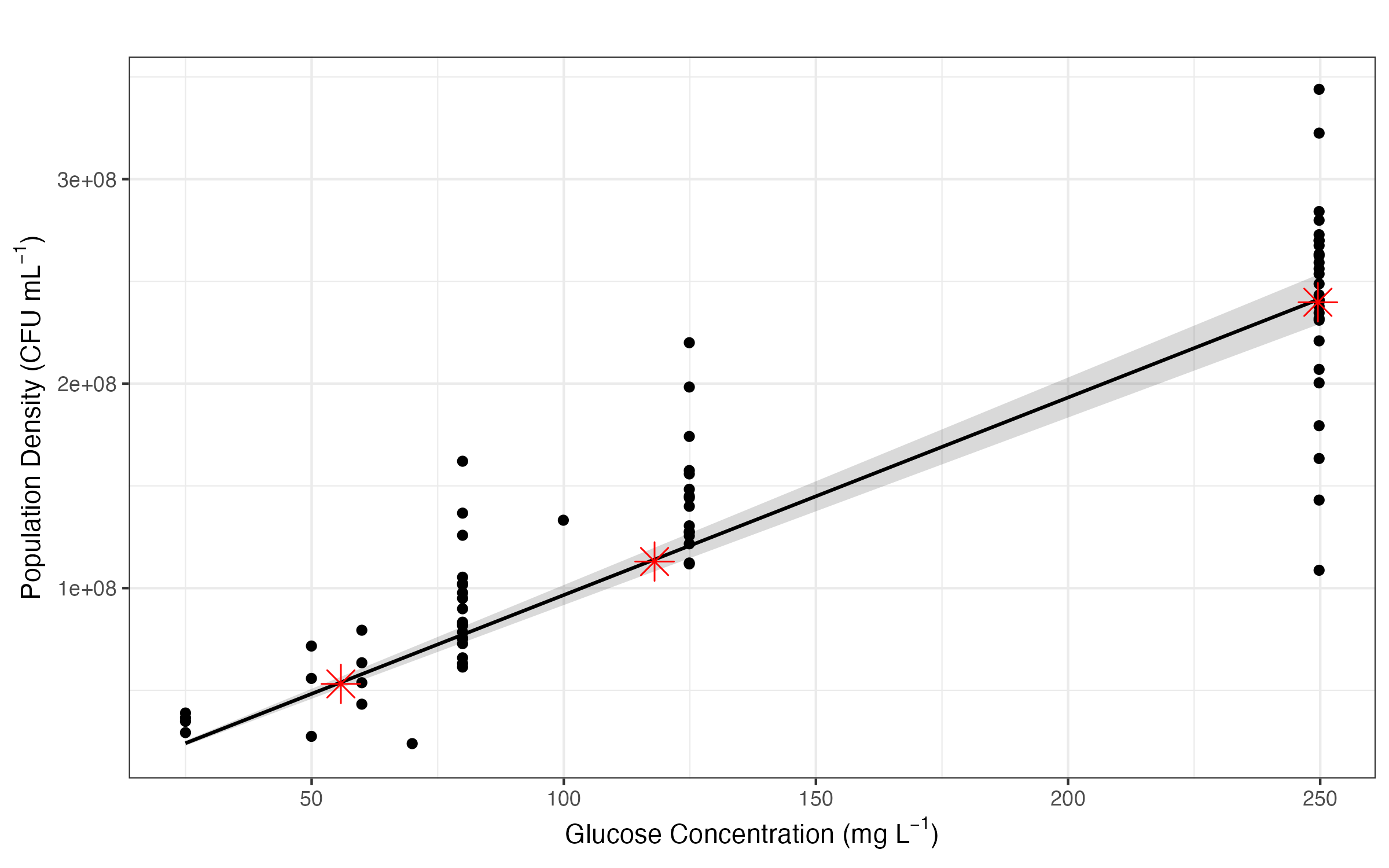

Supplement: S14 Fig — Used to fit parameter Met1 in ODE models. Black points show published lab data from [3] on population density and glucose provision in E. coli MG1655 used to fit this regression. Black line and shaded area show fitted relationship and 95% confidence interval, respectively, of a mixed effects model accounting for random effects of experimental block and plate. Red stars show output, in final population density, from initial ODE model A under differing initial glucose concentrations. Raw data for fitting can be found in S13 Data, data from ODE output can be found in S1 Data. (TIFF) [file pbio.3002711.s014.tiff]

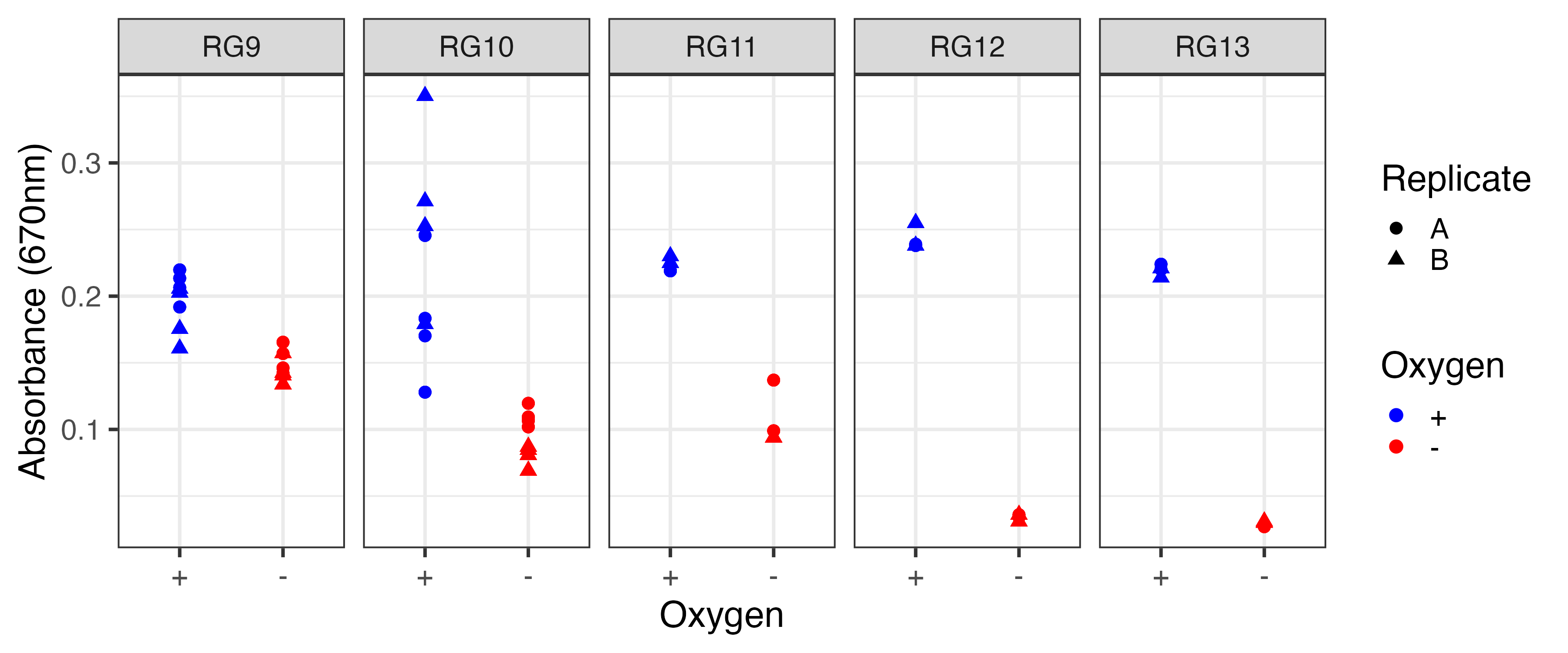

Supplement: S15 Fig — Each of 5 blocks is shown as a separate facet; within each block 2 sets of paired fluctuation assays (A and B) were conducted in aerobic and anaerobic conditions, for each of these sets 2–4 measurements of resorufin/dihydro resorufin absorbance were taken after 24 h of growth. Raw data can be found in S5 Data. (TIFF) [file pbio.3002711.s015.tiff]

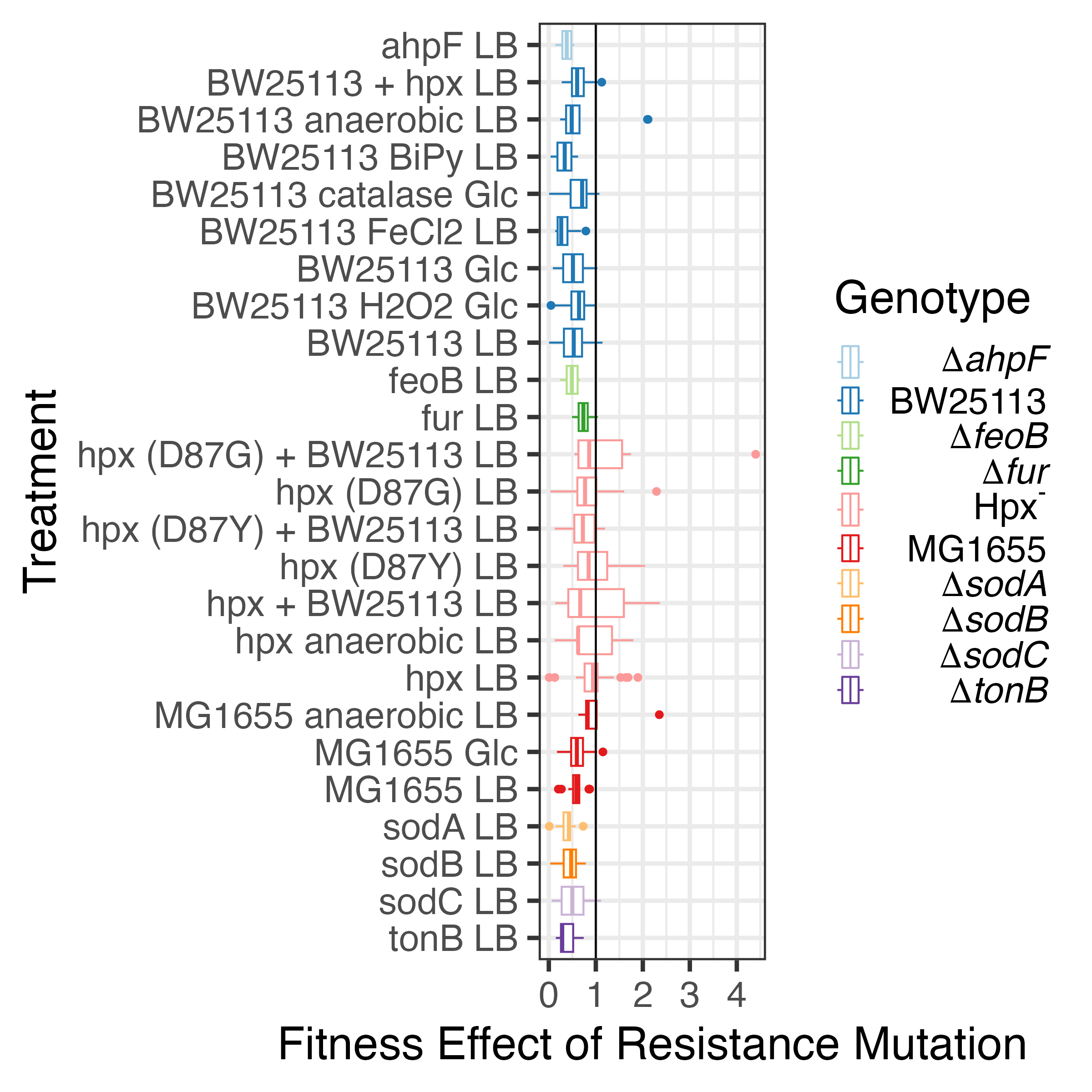

Supplement: S16 Fig — Boxplots shown for each treatment with colour representing genotype. Vertical lines inside boxes represent the median for that treatment, with the boxes depicting the interquartile range. The black vertical line at a fitness effect size of 1 represents neutral fitness effects. This data is used to fit regression 3. Raw data can be found in S14 data. (TIFF) [file pbio.3002711.s016.tiff]

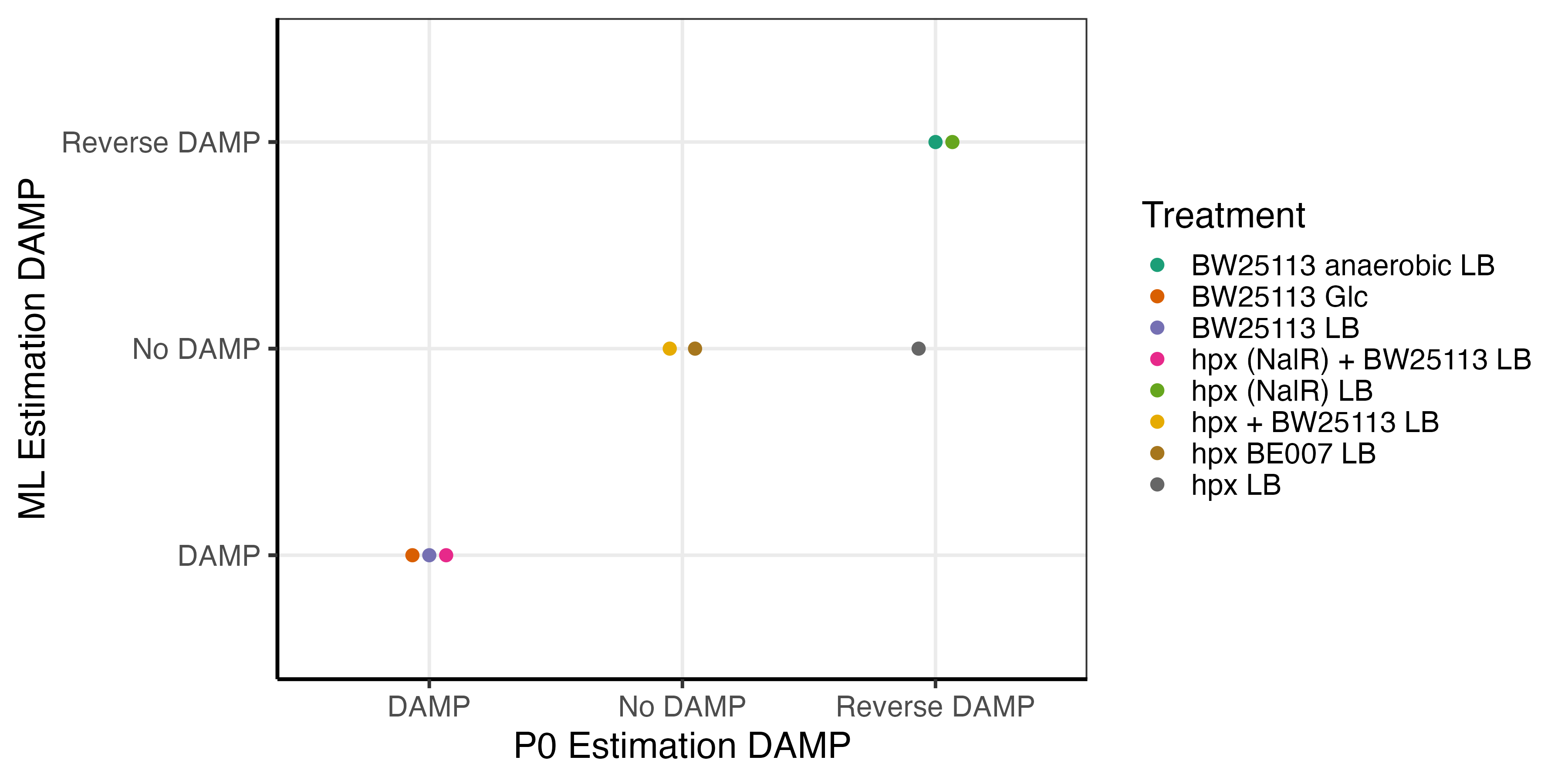

Supplement: S17 Fig — Treatments in which 8 or more fluctuation assays can be analysed by the p0 method are shown. Colour indicates treatment identity. The only treatment to change category is Hpx- strain LC106 which moves from “no DAMP” with ML estimates to “reverse DAMP” with p0 estimates; this does not refute our conclusions that Hpx-strains display no negative association between mutation rate and population density. All Hpx- points are strain LC106 unless indicated as BE007. Raw data used can be found in S5 Data. (TIFF) [file pbio.3002711.s017.tiff]

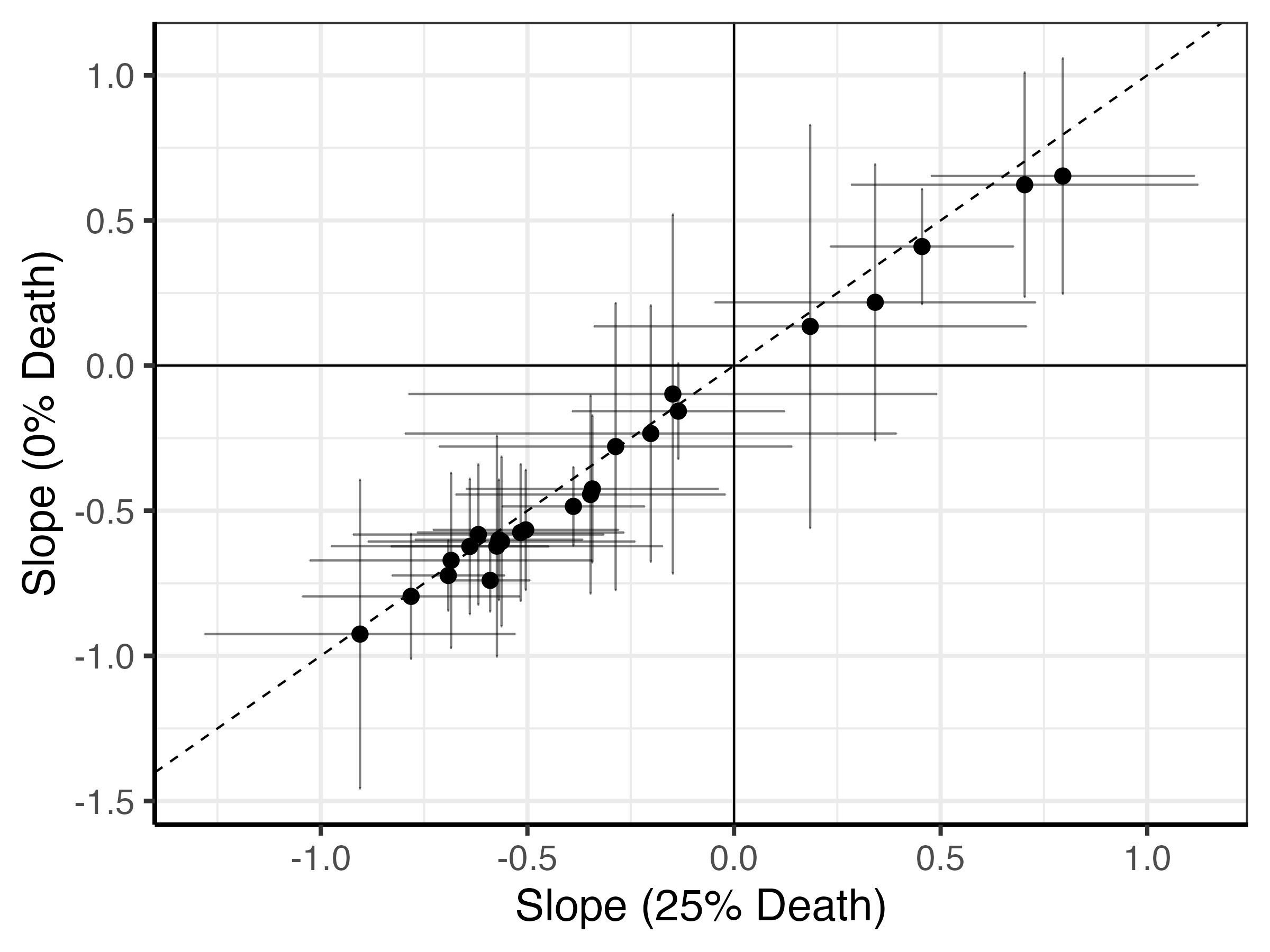

Supplement: S18 Fig — Solid lines indicate a slope of 0 (no DAMP), dashed line shows identical slope values for both estimates. All treatments remain in the same category (DAMP, no DAMP, or reverse DAMP). Raw data used can be found in S5 Data. (TIFF) [file pbio.3002711.s018.tiff]
