# Supplementary material for: Collective peroxide detoxification determines microbial mutation rate plasticity in E. coli
Source: PLoS Biol. 2024 Jul 15;22(7):e3002711. doi: 10.1371/journal.pbio.3002711 (PMC11272383; doi:10.1371/journal.pbio.3002711)
Supplement: S1 Supplementary Statistics — (PDF) [file pbio.3002711.s024.pdf]

Supplementary Text - Statistical Models and Tests for Green et al., 2024

Regression 1 (Fig. 2)

Simulated mutation rates across 5 population densities in ODE models A-K (with $\log_2$ transformation) are fitted as a function of population density (with $\log_2$ transformation).

ANOVA table for Regression 1

	Df	*Sum Sq*	*Mean Sq*	*F value*	*Pr(>F)*
log2(wt)	1	5.39	5.39	1840	1.54E-30
model	10	0.388	0.0388	13.3	6.35E-09
log2(wt):model	10	19.4	1.94	665	4.66E-35
Residuals	33	0.0965	0.00292		

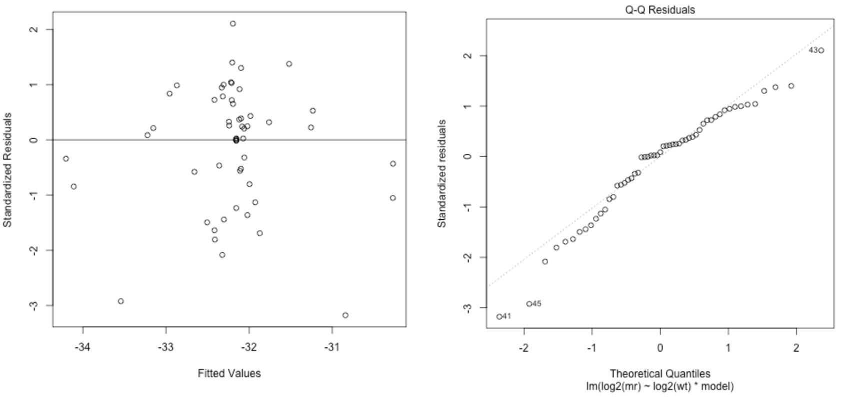

Diagnostic plots for Regression 1.
Standardised residuals by fitted values and normal quantile-quantile plot of standardised residuals.

Model	Slope	CI_95	Intercept	Intercept_95
A	0.0912	0.016	-34.8	0.447
B	-0.0121	0.0161	-31.7	0.449
C	-0.00144	0.016	-32.1	0.447
D	-0.906	0.016	-7.01	0.447
E	0.0868	0.016	-34.5	0.447
F	-0.885	0.016	-7.57	0.447
G	-0.276	0.016	-24.7	0.447
H	0.0806	0.016	-34.6	0.447
I	-0.623	0.016	-14.9	0.447
J	0.104	0.016	-35.1	0.447
K	0.096	0.016	-34.9	0.447

Table of coefficients for slopes fitted by regression 1 95% CI are given by Slope ± CI_95 for slope and for intercept by Intercept ± Intercept_95.

Regression 2 (Fig. 2a)

Mutation rates (with log_2 transformation) of the wildtype BW25113 (ancestor) strain grown in minimal glucose media are fitted as a function of population density (also with log_2 transformation). Random effects of experimental plate (31 levels) within experimental block (12 levels) on the intercept are included. This model uses a subset of the data used in Regressions 3-4.

ANOVA for Regression 2

	numDF	*denDF*	*F-value*	*p-value*
(Intercept)	1	19	67541.4	0
Log2(D_genotype)	1	16	148.7	1.627e-09

Variance and Standard Deviations for Random Effects for Regression 2

	Variance
block	1.211768e-01
plate_ID	1.655934e-09
Residual	1.968184e-01

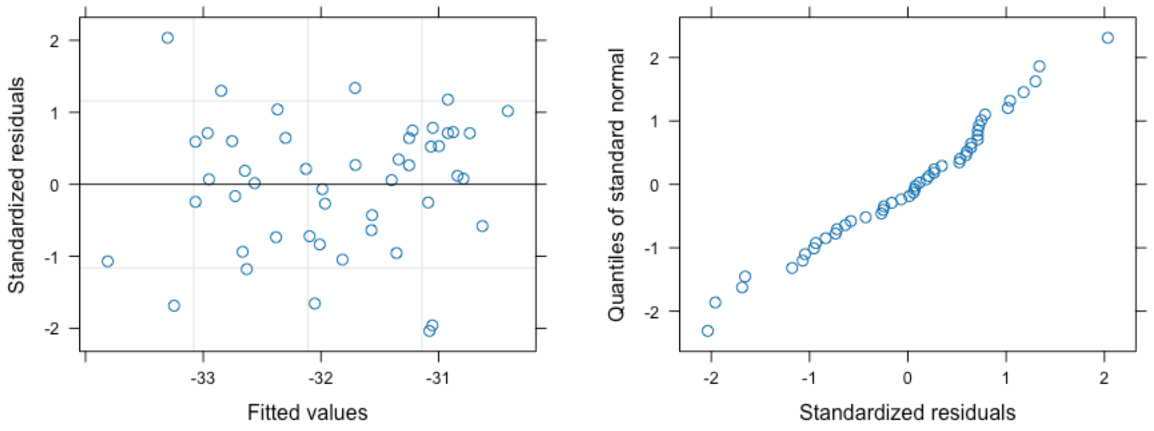

Diagnostic plots for Regression 2.
Standardised residuals by fitted values and normal quantile-quantile plot of standardised residuals.

Regression 3 – (Fig S16)

The model fitting the fitness effect of resistance during fluctuation assays is the $\log_2$ transformed ratio of wt:mutant fitness as a function of the treatment (genotype, coculture strain and growth conditions; 48 levels). Random effects of experimental plate (165 levels) nested within experimental block (59 levels) nested within experimenter (5 levels), each affecting the intercept, are also fitted.

ANOVA for Regression 3

	numDF	*denDF*	*F-value*	*p-value*
(Intercept)	1	508	32.1	2.47E-08
Treatment	47	508	3.14	1.75E-10

Variance and Standard Deviations of Random Effects for Regression 3

	Variance	*StdDev*
Experimentor..	1.53E-01	3.91E-01
block..	1.69E-11	4.11E-06
Plate	2.13E-08	1.46E-04
Residual	1.02E+00	1.01E+00

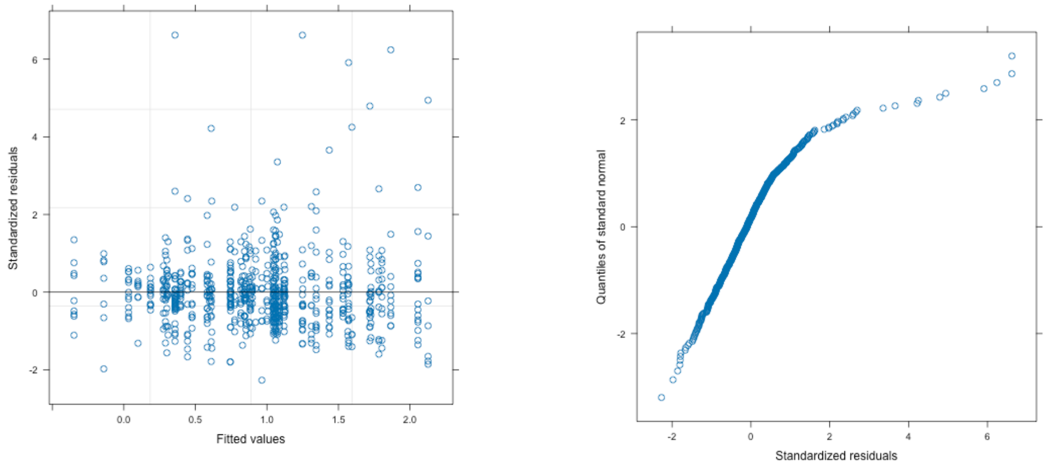

Diagnostic plots for Regression 3.
Standardised residuals by fitted values and normal quantile-quantile plot of standardised residuals.

Regression 4 (Fig. 3-4, Table S1, Fig. S4,5,8,9,11,17,18)

The model shown in fig. 3 and fig. 4 fits log_2 mutational events per mL against mean-centred log_2 density (Dc) allowing for differences in intercept and slope among the 48 treatments. Random effects of experimental plate (155 levels) nested within experimental block (57 levels) nested within experimenter (5 levels), each affecting the intercept, are also fitted. A series of variants of this model was constructed allowing differences in variance (i.e. heteroscedasticity) associated with one or two covariates.

Potential variance covariates considered were: experimental block, genotype, external treatment (e.g. anaerobiosis or chelator), date, experimental plate, selective marker (i.e. rifampicin or nalidixic acid), nutrient type, nutrient concentration and experimenter identity, all treated as discrete effects with a different variance at each level. Continuous variance covariates allowed variance to change as a power function of the covariate. Potential continuous variance covariates considered were: the fitted values of the response variable, the percentage of LB in the media, the concentration of glucose in the media, the initial population size (N0), the range between 95% confidence intervals on the mutation rate, the average final volume of the parallel cultures, density (estimated by colony forming units), the number of generations (log(final population)-log(initial population))/(log(2)), the estimated number of mutational events (m), the lower and upper bounds and standard deviation in that estimate, the estimated fitness ratio of cells with to without the selective marker, the mutation rate (m/final population size), the upper and lower bounds on the mutation rate, the number of parallel cultures, the final population size, the standard variation and coefficient of variation in that estimate, the ratio of final to initial population sizes, the initial and final weight of the experimental plate and the incubation time of the experimental plate.

The model variant with the lowest AIC was then chosen. In this case the model allowed variance to change with the upper bound of m and with genotype. The two slopes and intercepts of hpx^{-}_{nalR} strains D87Y and D87G were very similar, and this model was therefore further simplified (improving AIC further) by combining these effects to estimate single intercept and slope values for these strains. Treatments with these hpx^{-}_{nalR} strains combined are termed 'TreatmentHC'.

ANOVA table for Regression 4

	numDF	*denDF*	*F-value*	*p-value*
(Intercept)	1	469	79	0
Dc	1	469	1760	0
TreatmentHC	45	469	38.8	0
Dc:TreatmentHC	45	469	7.48	0

Variance and Standard Deviations of Random Effects for Regression 4

	Variance
Experimentor..	0.34346788
block..	0.07354267
plate_ID..	0.01168155
Residual	0.5680057

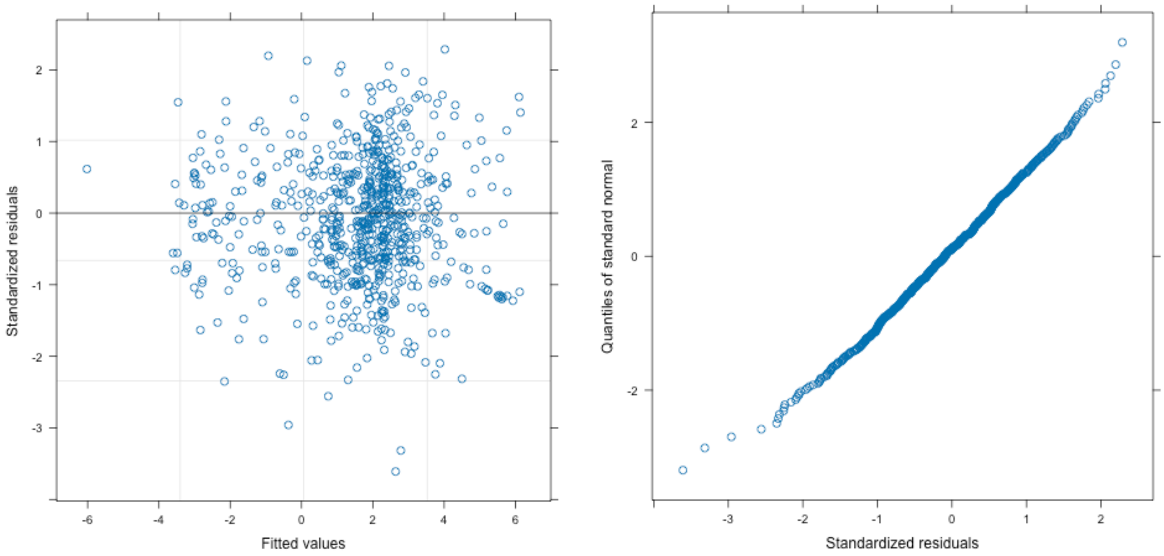

Diagnostic plots for Regression 4.
Standardised residuals by fitted values and normal quantile-quantile plot of standardised residuals.

Regression 5

To estimate hydrogen peroxide concentration from arbitrary fluorescence units (AFU) in the amplex ultra-red assay, a standard curve was fitted with known hydrogen peroxide concentrations (raw data shown below). H_2O_2 added in the standard is fitted as a function of net fluorescence (final fluorescence minus initial fluorescence or fluorescence of a 0-concentration standard). A random effect of block affecting the intercept and slope (NetFU) was also included.

ANOVA table for Regression 5

	numDF	*denDF*	*F-value*	*p-value*
(Intercept)	1	112	254	0
NetFU	1	112	90.3	4.44E-16

Variance and Standard Deviations and Covariance for Random Effects for Regression 5

	Variance	*Corr*
(Intercept) Block	1.45E+00	(Intr)
NetFU	5.84E-09	0.75
Residual	6.19E-01	

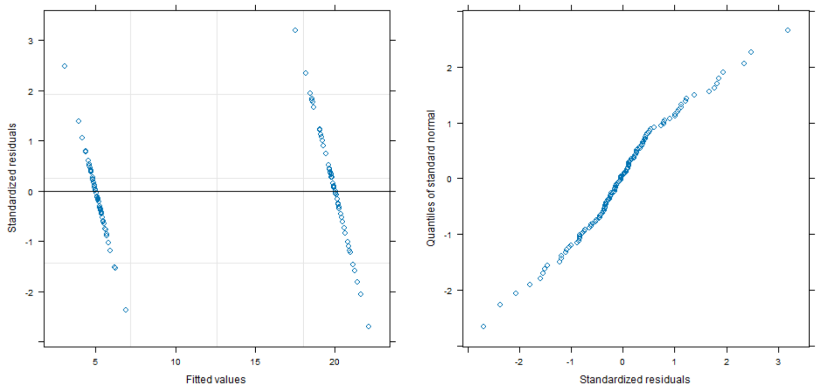

Diagnostic plots for Regression 5.
Standardised residuals by fitted values and normal quantile-quantile plot of standardised residuals.

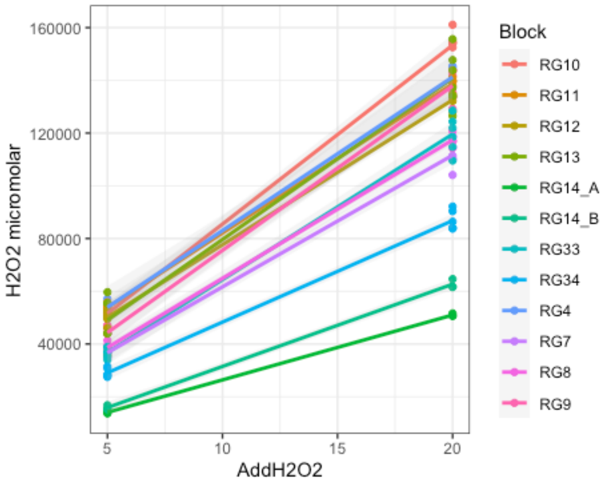

Raw data used to fit regression 5. NetFU calculated as AFU after addition of horseradish peroxidase – AFU prior to HRP addition. H_2O_2 diluted to known concentrations from a stock solution of 3% (v/v). Lines coloured by experimental block.

Regression 6 – Fig. S7B

Predicted H_2O_2 (in µM units) using regression 5 is fitted as a function of nutrient type, nutrient level and presence or absence of cells. Initially all interactions between effects were included with the three-way interaction between all effects and the interaction between nutrient type and nutrient level then removed sequentially, each improving the fit of the model (lower AIC). Random effects on the intercept of reaction tube nested within block (labelled as 'replicate' in Fig. S7A) nested within week are also included.

ANOVA table for Regression 6

	numDF	*denDF*	*F-value*	*p-value*
(Intercept)	1	120	41.80638	2.27E-09
Nutrient	1	46	0.868673	0.356189
Strain	1	46	63.42915	3.34E-10
NutLev	1	46	0.032108	0.858579
Nutrient:Strain	1	46	3.61737	0.063452
Strain:NutLev	1	46	9.801188	0.003028

Variance and Standard Deviations for Random Effect for Regression 6

	Variance
Week..	4.15E+00
Block..	8.15E-08
Tube..	2.01E+00
Residual	4.67E-02

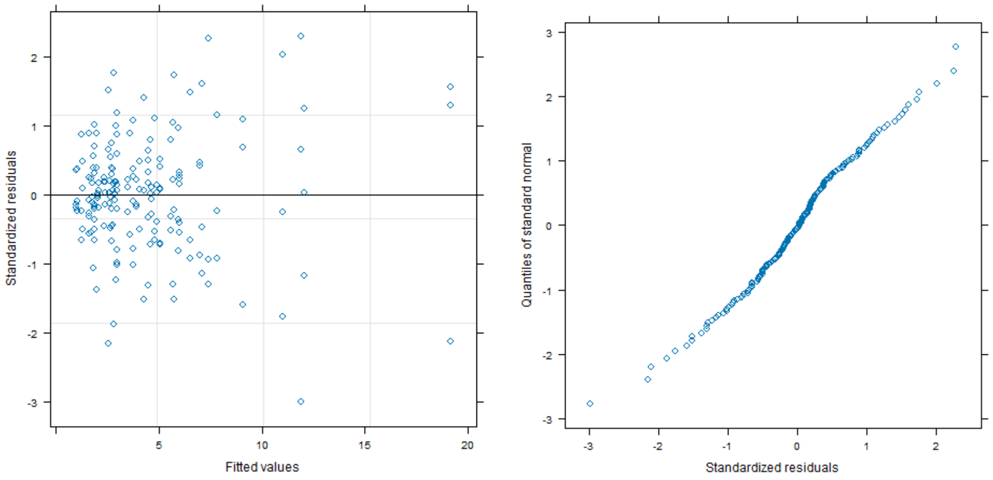

Diagnostic plots for Regression 6.
Standardised residuals by fitted values and normal quantile-quantile plot of standardised residuals.

Regression 7 – Fig. S7A

Log_2 predicted H_2O_2 (in µM units) using regression 5 is fitted as a function of log_2 cell density (estimated by absorbance at 600nm) and nutrient type (Rich or Minimal). Initially interaction between the effects was included (reg 7A) this was then removed along with the effect of nutrient type, improving the fit of the model (reg 7B). Random effects on the intercept of reaction tube nested within block (referred to as 'Replicate in Fig. S7A) were included.

ANOVA for Regression 7A

	numDF	*denDF*	*F-value*	*p-value*
(Intercept)	1	72	35.0611502	1.00E-07
log2(OD_bc)	1	26	33.2684042	4.49E-06
Nutrient	1	26	0.02762148	0.86928753
log2(OD_bc):Nutrient	1	26	0.000918	0.97606041

ANOVA for Regression 7B

	numDF	*denDF*	*F-value*	*p-value*
(Intercept)	1	72	35.2	9.61E-08
log2(OD_bc)	1	28	35.8	1.91E-06

Variance and Standard Deviations for Random Effects for Regression 7B

	Variance
Block..	0.35967362
Tube..	0.08204864
Residual	0.01005952

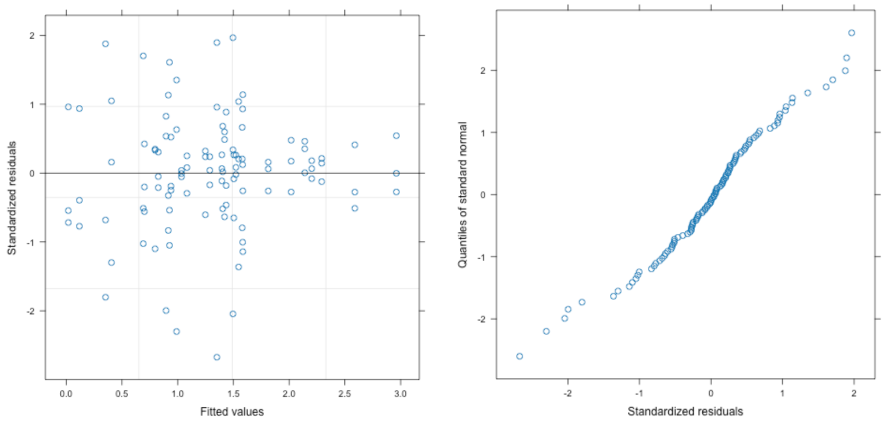

Diagnostic plots for Regression 7B

Standardised residuals by fitted values and normal quantile-quantile plot of standardised residuals.

Regression 8 – Fig. S10

Regression 8 fits log2 mutation rate of the hpx^{-}_{nalR} strains against mean-centred log2 total density (estimated via CFU) initially allowing for differences in intercept and slope among the four treatments (different strain/coculture combinations). Combining the two hpx^{-}_{nalR} strains significantly improves the fit of the model (as with regression 4) and so only the effect of coculture on intercept and slope is used. Random effects of experimental plate nested within experimental block on the intercept are included.

ANOVA for Regression 8

	numDF	*denDF*	*F-value*	*p-value*
(Intercept)	1.00E+00	29	1090	0.00E+00
log2(D_total)	1.00E+00	29	57.2	2.43E-08
coculture	1.00E+00	2.90E+01	16.3	0.000363
log2(D_total):coculture	1.00E+00	2.90E+01	14.4	0.000704

Variance and Standard Deviations for Random Effects for Regression 8

	Variance
Block	1.81E-10
plate_ID	1.41E-10
Residual	5.68E-01

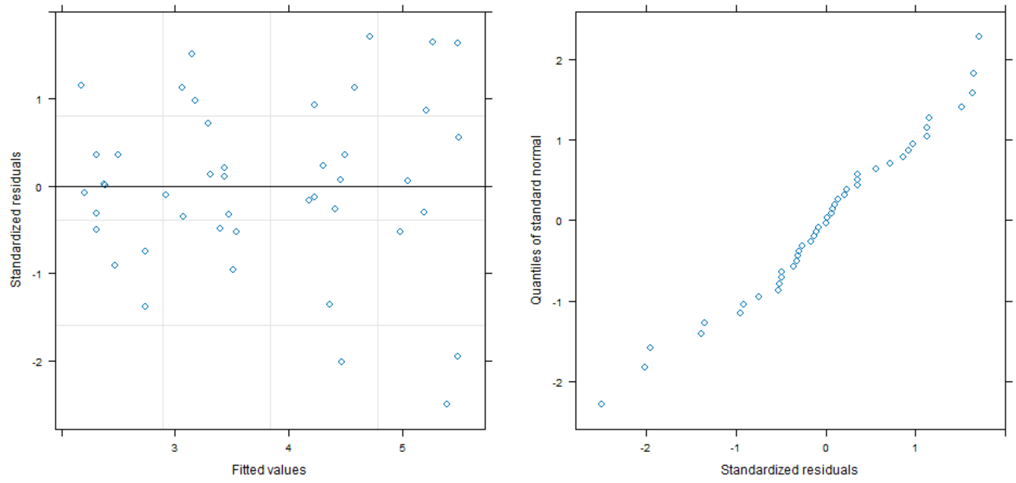

Diagnostic plots for Regression 8.
Standardised residuals by fitted values and normal quantile-quantile plot of standardised residuals.

Regression 9A – Fig. S12

Regression 9 fits the plating efficiency of the $hpx^{-}_{nalR\&rifR}$ strains used in a reconstruction test as a function of the plating environment, strain identity and the interaction of environment and strain, the random effect of the 6-well agar plate on which the cultures were plated is also included. Regression 9B is identical but the 4 cell treatments (hpx^{-} and wt low high and medium density) are combined to one level and Treatment renamed as CombT.

ANOVA for Regression 9A

	numDF	*denDF*	*F-value*	*p-value*
(Intercept)	1	68	1090	0
Treatment	4	68	37	2.22e-16
Strain	1	68	59.5	7.32e-11
Treatment:Strain	4	68	3.26	1.66e-02

Variance and standard deviations for random effects for Regression 9A

	Variance	*StdDev*
(Intercept)	0.005348034	0.07313026
Residual	0.023882641	0.15454010

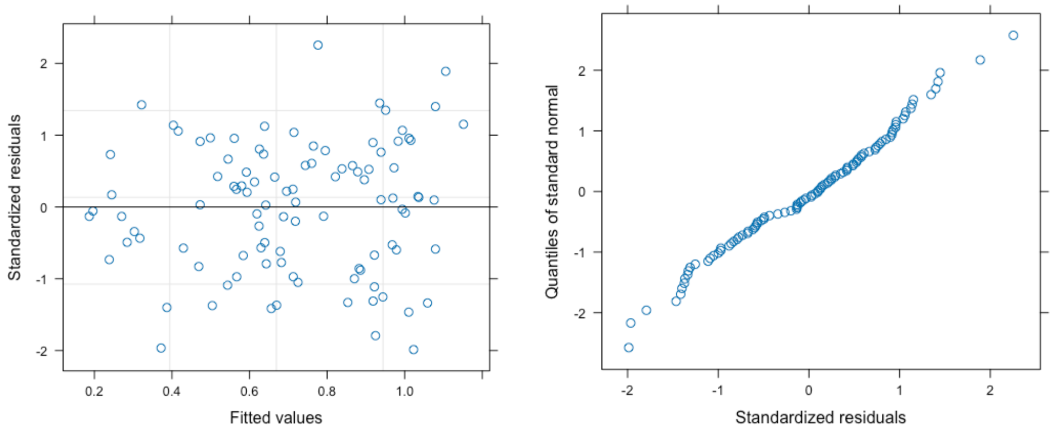

Diagnostic plots for Regression 9A.
Standardised residuals by fitted values and normal quantile-quantile plot of standardised residuals.

ANOVA for Regression 9B

	numDF	*denDF*	*F-value*	*p-value*
(Intercept)	1	74	1130	0
CombT	1	74	135	0
Strain	1	74	58.1	6.72e-11
CombT:Strain	1	74	1.88	0.174

Variance and standard deviations for random effects for Regression 9B

	Variance	*StdDev*
(Intercept)	0.004528522	0.0672943

Residual	0.025828870	0.1607136

ANOVA comparing Regression 9A to Regression 9B

	Model	*df*	*AIC*	*BIC*	*logLik*	*Test*	*L.Ratio*	*p-value*
Regression 9A	1	12	-60.6	-29.3	42.3			
Regression 9B	2	6	-60.9	-45.3	36.5	1 vs 2	11.7	0.0696
