# Supplementary material for: Collective peroxide detoxification determines microbial mutation rate plasticity in E. coli
Source: PLoS Biol. 2024 Jul 15;22(7):e3002711. doi: 10.1371/journal.pbio.3002711 (PMC11272383; doi:10.1371/journal.pbio.3002711)
Supplement: S1 Appendix — (DOCX) [file pbio.3002711.s025.docx]

Appendix 1 - ODE Model Variants

This appendix expands on the ODE models presented in the Results and Methods sections of “*Working together to control mutation: how collective peroxide detoxification determines microbial mutation rate plasticity*” Green et al.

[Figure A1: Array of flow diagrams illustrating changes introduced by model variants B-K. 3](#_Toc164441090)

[Figure A2: All variable dynamics over time with initial glucose 250mg/L in model A 4](#_Toc164441091)

[Figure A3: Mutation rate dynamics in all ODE variants 5](#_Toc164441092)

[Figure A4: DAMP slope during exponential phase in all variants. 6](#_Toc164441093)

[Figure A5: Decreasing permeability results in reduced mutation rate and shallower DAMP slope in model D 7](#_Toc164441094)

[Figure A6: Model B – Glucose uptake rate is inversely related to external glucose concentration. 8](#_Toc164441095)

[Figure A7: Model C – Fixed concentration of internal ROS 8](#_Toc164441096)

[Figure A8: Model D – Constant rate of external ROS production with diffusion across the membrane. 9](#_Toc164441097)

[Figure A9: Model E – ROS removal rate (ahp/kat degradation) increases with internal glucose concentration. 10](#_Toc164441098)

[Figure A10: Model F – ROS removal rate (ahp/kat degradation) increases with cell density 10](#_Toc164441099)

[Figure A11: Model G – odGTP degradation rate (MutT activity) depends on internal glucose concentration 11](#_Toc164441100)

[Figure A12: Model H – odGTP degradation rate (MutT activity) depends on odGTP concentration 11](#_Toc164441101)

[Figure A13: Model I – odGTP degradation rate (MutT activity) depends on internal ROS concentration 12](#_Toc164441102)

[Figure A14: Model J – odGTP degradation rate (MutT activity) shows Michaelis Menten saturating kinetics. 12](#_Toc164441103)

[Figure A15: Model K – Diffusion of ROS across the cell membrane and Michaelis Menten kinetics of AhpCF/KatEG activity are included however no external ROS production is included. 13](#_Toc164441104)


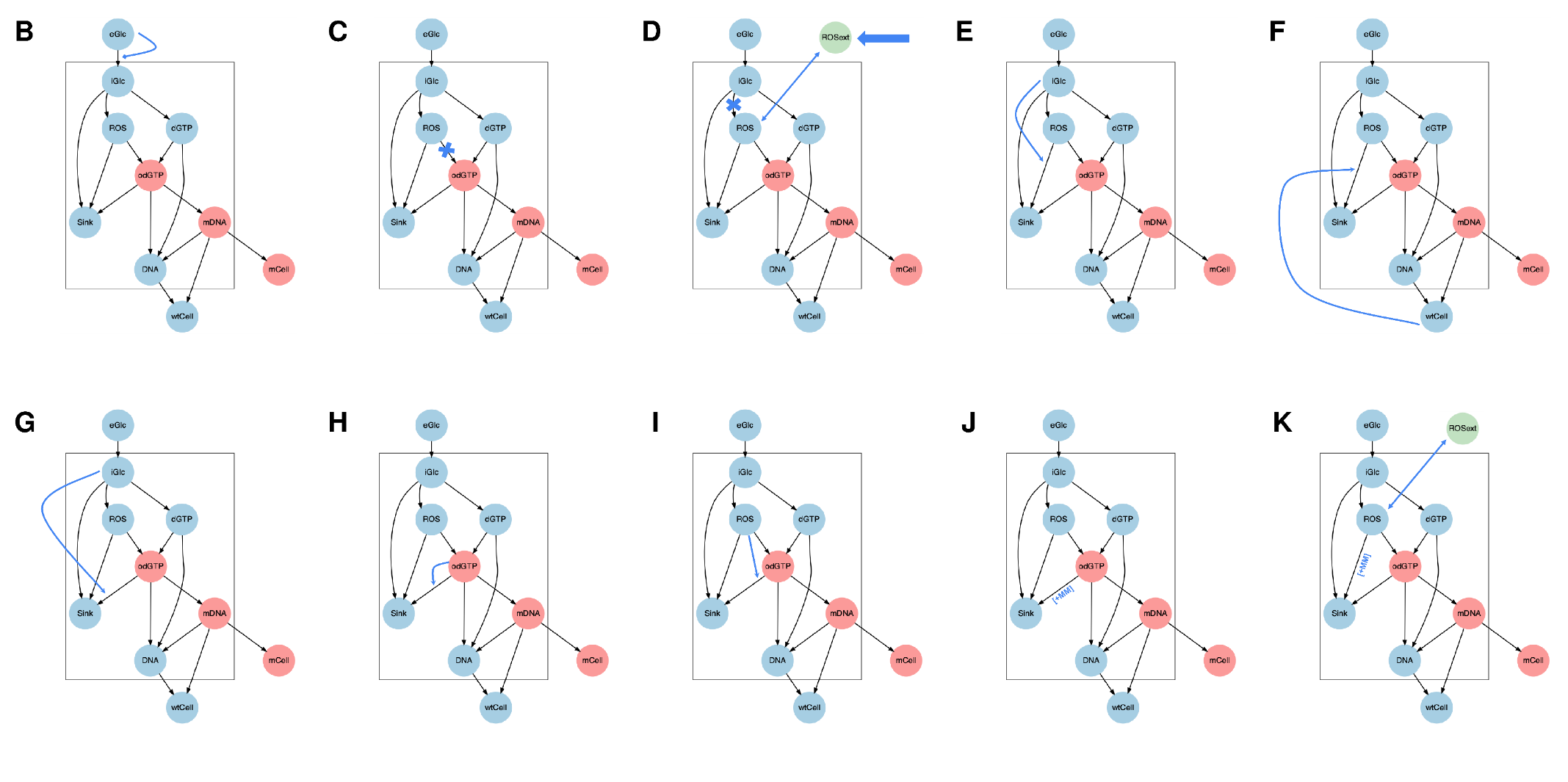


Figure A1: Array of flow diagrams illustrating changes introduced by model variants B-K. Each illustration shows a flow diagram representing ODE model A with a square enclosing those variables existing within the cytoplasm. Changes introduced by each variant are drawn on top. B: Glucose uptake rate downregulated by eGlc; C: Rate of dGTP oxidation depends only on dGTP and constants ROSC & O2; D: Constant ROS production in the environment diffuses through the membrane and ROS production from iGlc is removed; E: Rate of ROS degradation upregulated by iGlc; F: Rate of ROS degradation upregulated by wtCell; G: Rate of odGTP degradation upregulated by iGlc; H: Rate of odGTP degradation upregulated by odGTP; I: Rate of odGTP degradation upregulated by ROS; J: Rate of odGTP degradation shows Michaelis Menten kinetics; K: Rate of ROS degradation shows Michaelis Menten kinetics and ROS is able to diffuse to and from the environment across the membrane.

Figure A2: All variable dynamics over time with initial glucose 250mg/L in model A
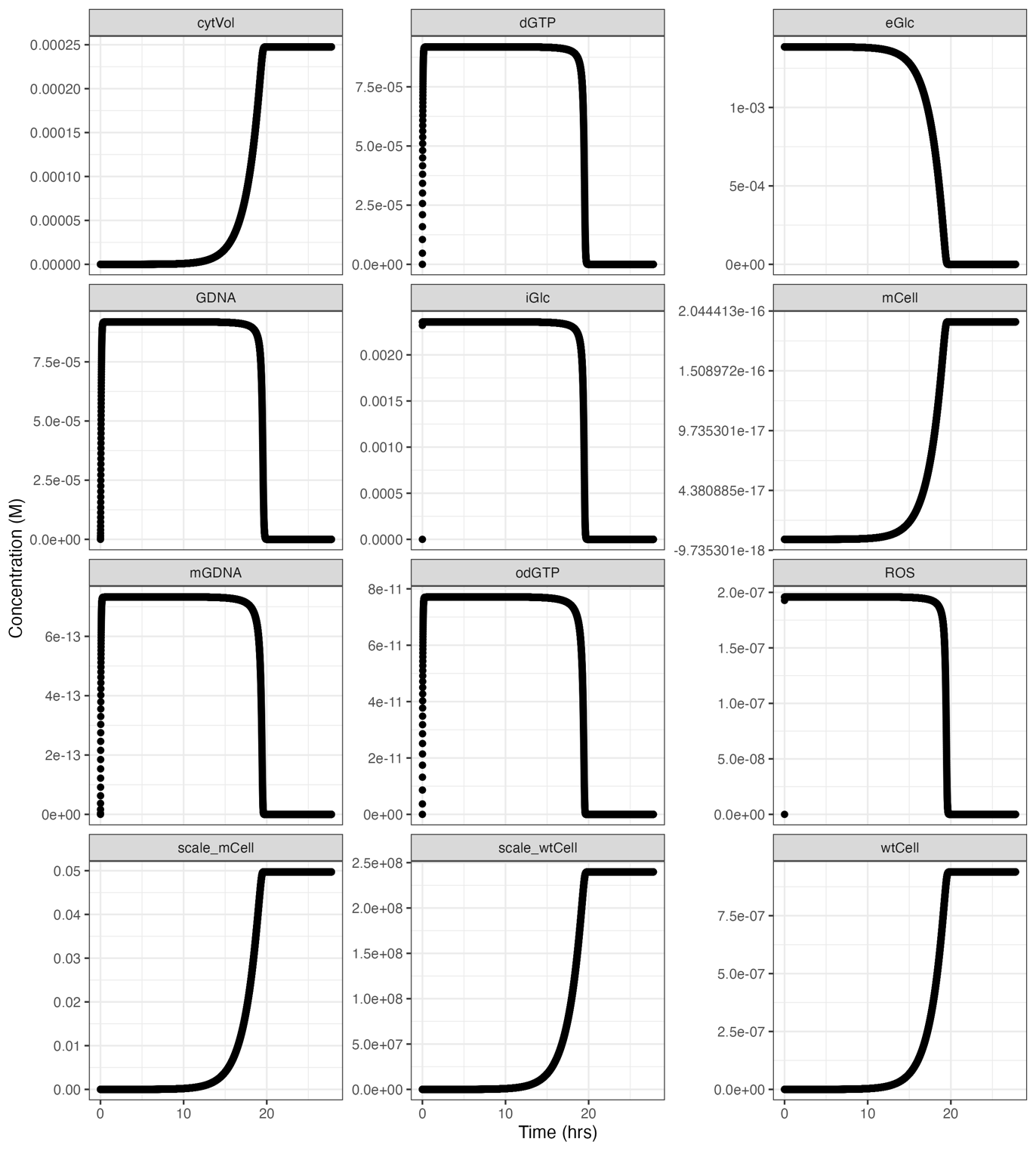


wtCell measures the molar concentration of GC nucleotide pairs in chromosomes in 1mL. scale_wtCell shows the population density in cells per mL. mCell and scale_mCell show the same measurements for incorrectly replicated GC basepairs causing mutations.

#

# Figure A3: Mutation rate dynamics in all ODE variants


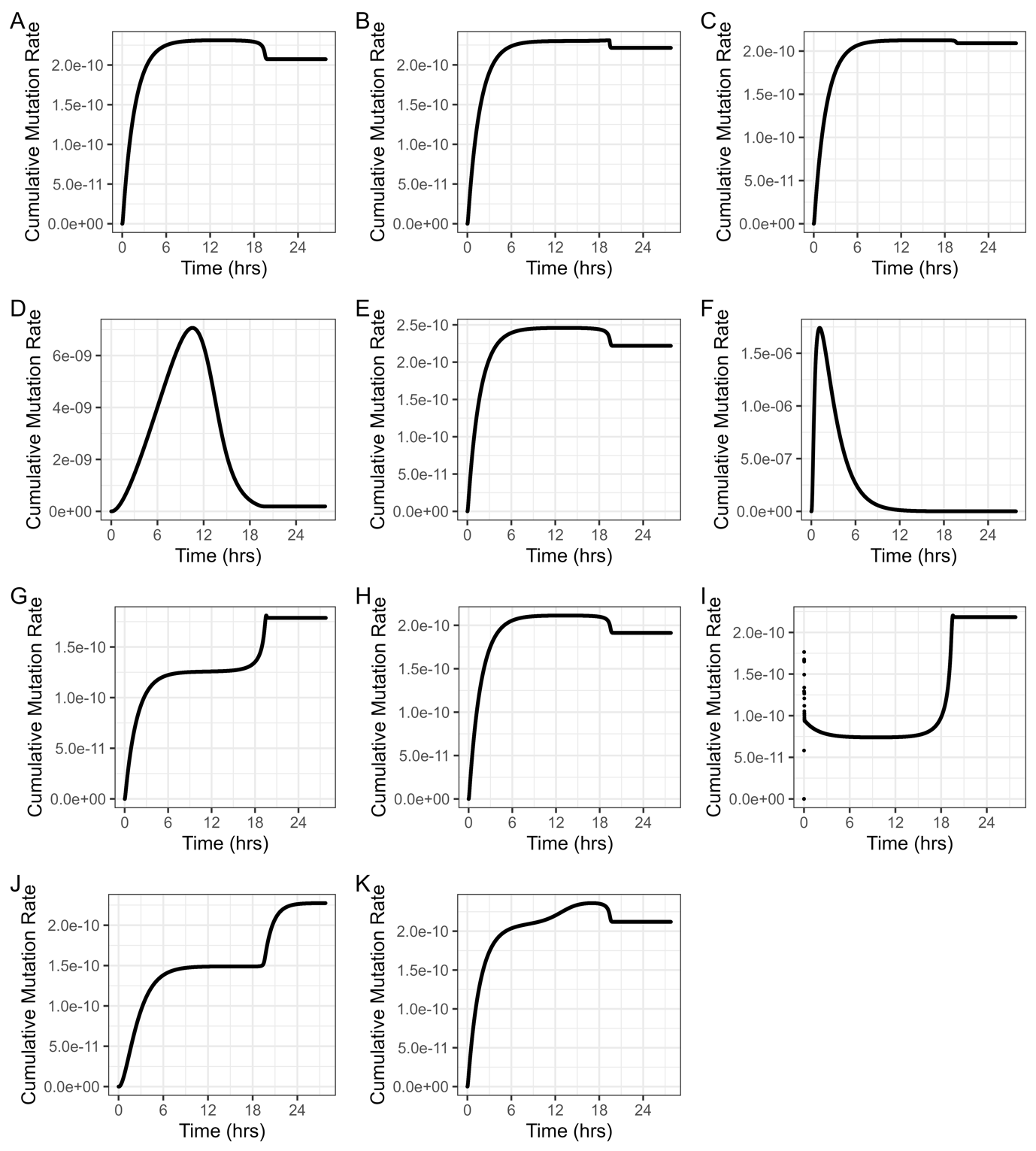


Cumulative mutation rate is defined as all mutant base pairs produced (mCell) divided by all base pairs produced (mCell+wtCell) at each time point. Plots A-K show dynamics for model variants A-K respectively all simulated at initial external glucose concentration of 250mg/L. Note that although models D and F appear to trend towards 0 both models reach a mutation rate of ~2x10^-10^ after 24 hours (see Fig. 2A).

# Figure A4: DAMP slope during exponential phase in all variants.


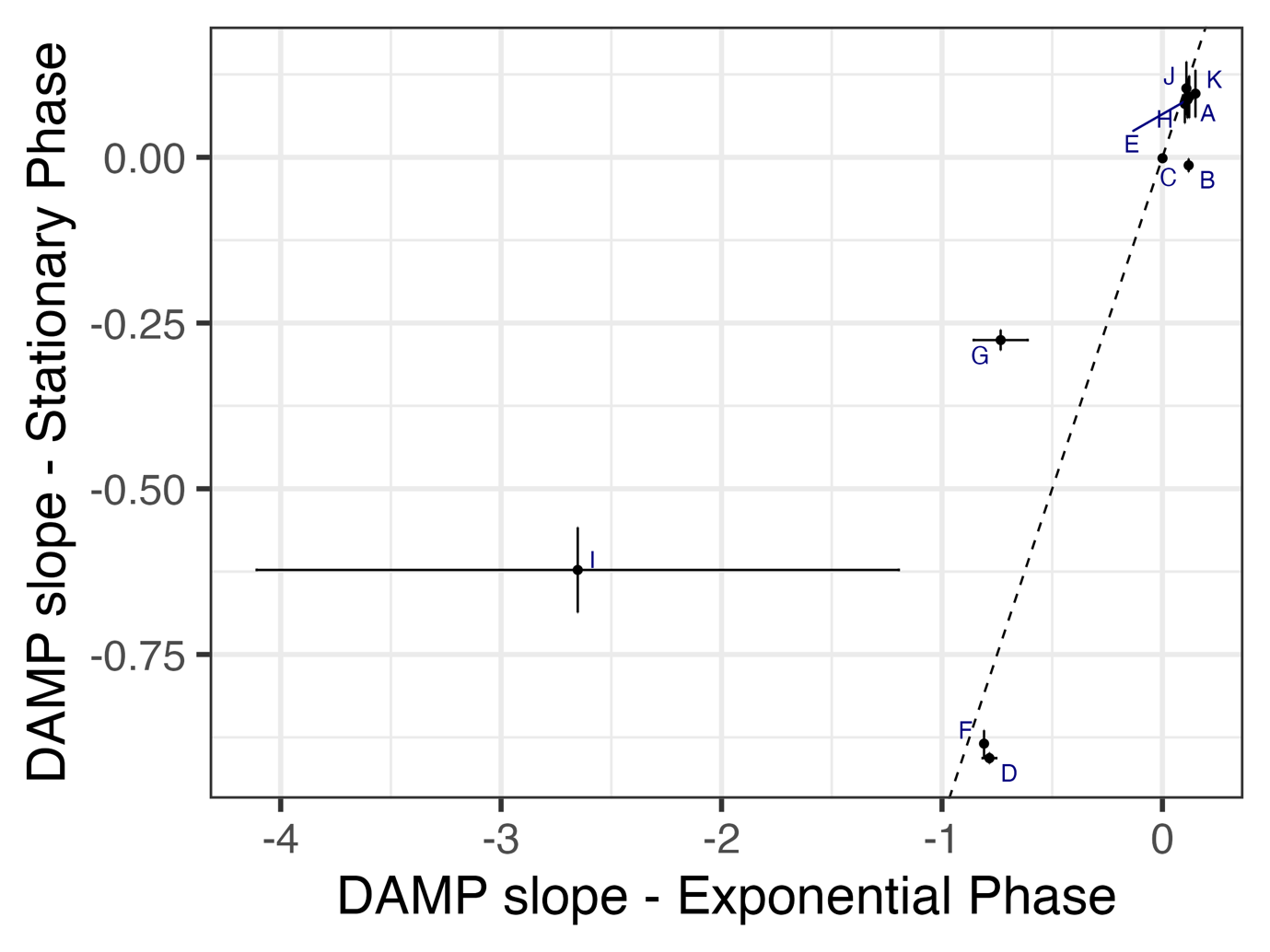


DAMP slope during exponential phase (16 hours growth) is plotted on the x axis and during stationary phase (1x10^5^ seconds = ~27.8hrs) is plotted on the y axis. The dashed line indicates equal values of DAMP at both time point. Model variants are labelled in blue. Error bars show 95% confidence intervals calculated as 1.96 x standard error.

DAMP slopes of all variants aside from G and I remain qualitatively unchanged regardless of growth phase. Models G and I have previously been shown to have less stable DAMP slope by the global sensitivity analysis (Fig.2B). Given that variants G and I still retain significantly negative slopes across both growth phases and are not the focus of the in culture experiments we conclude that measurements of ODE DAMP in the stationary phase are also representative of DAMP during exponential growth.

# Figure A5: Decreasing permeability results in reduced mutation rate and shallower DAMP slope in model D


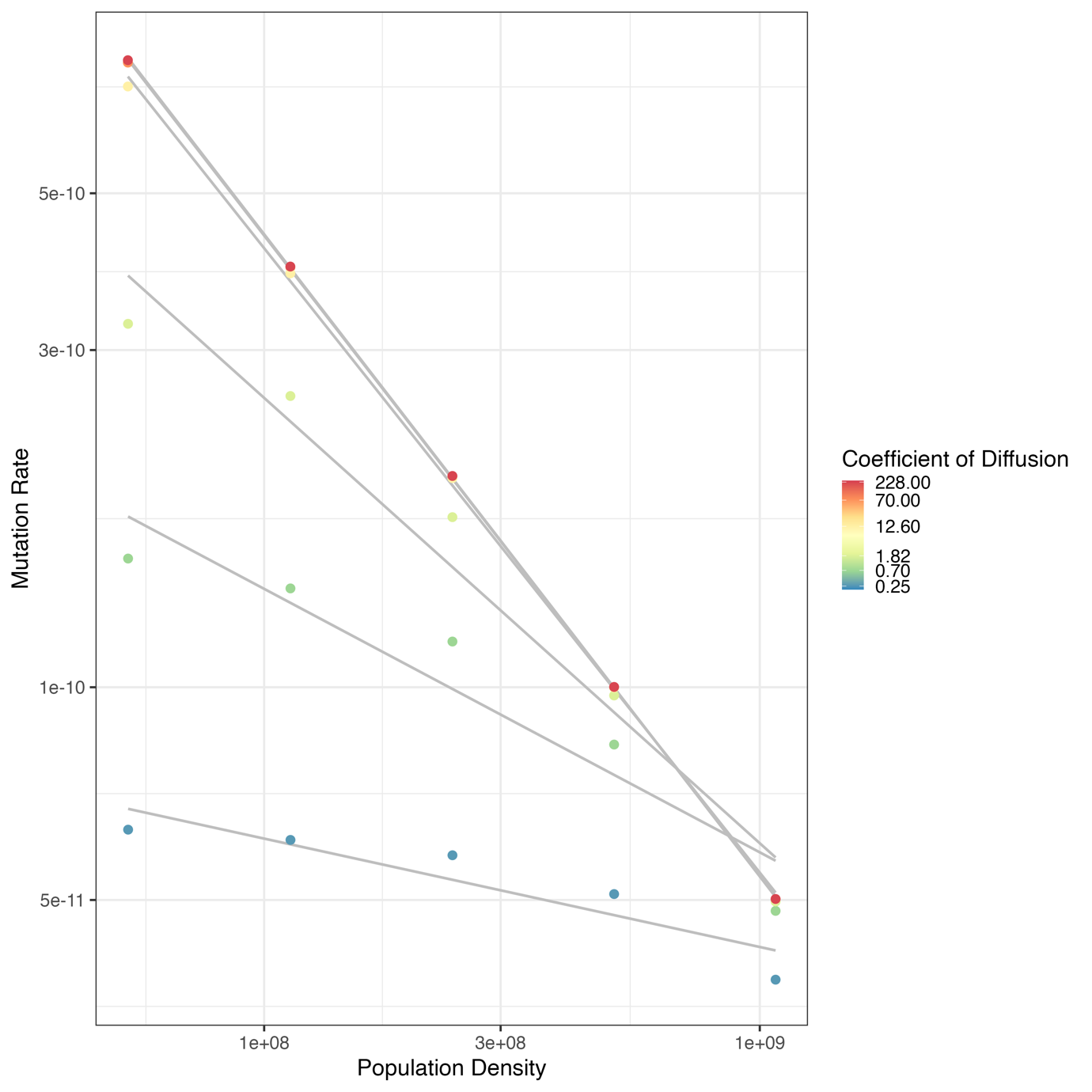


Mutation rate is shown per bp per generation, population density is shown in cells per mL. Note that points for 70 sit behind 228 points. Coefficient of diffusion for *E. coli* is 70 (1).

# Figure A6: Model B – Glucose uptake rate is inversely related to external glucose concentration.


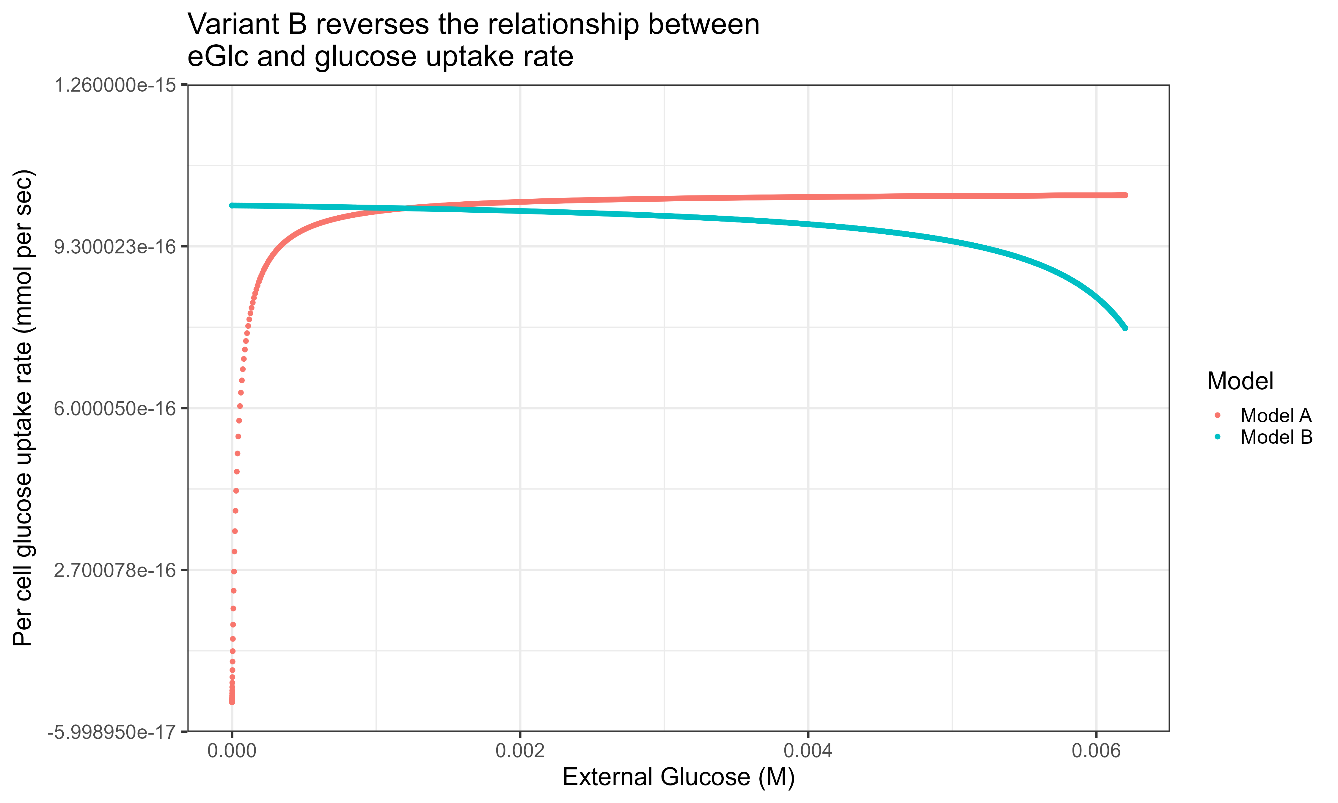


# Figure A7: Model C – Fixed concentration of internal ROS


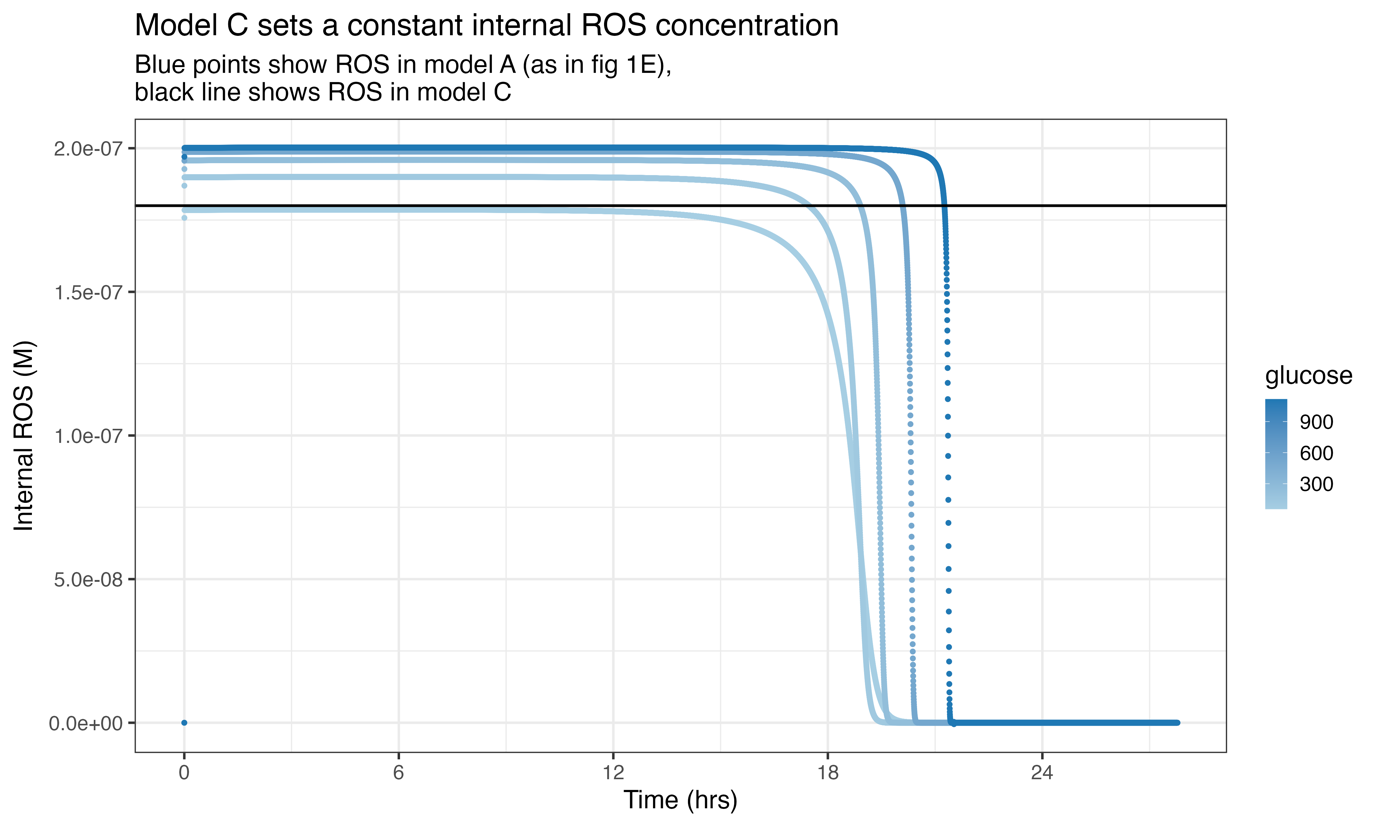


# Figure A8: Model D – Constant rate of external ROS production with diffusion across the membrane.


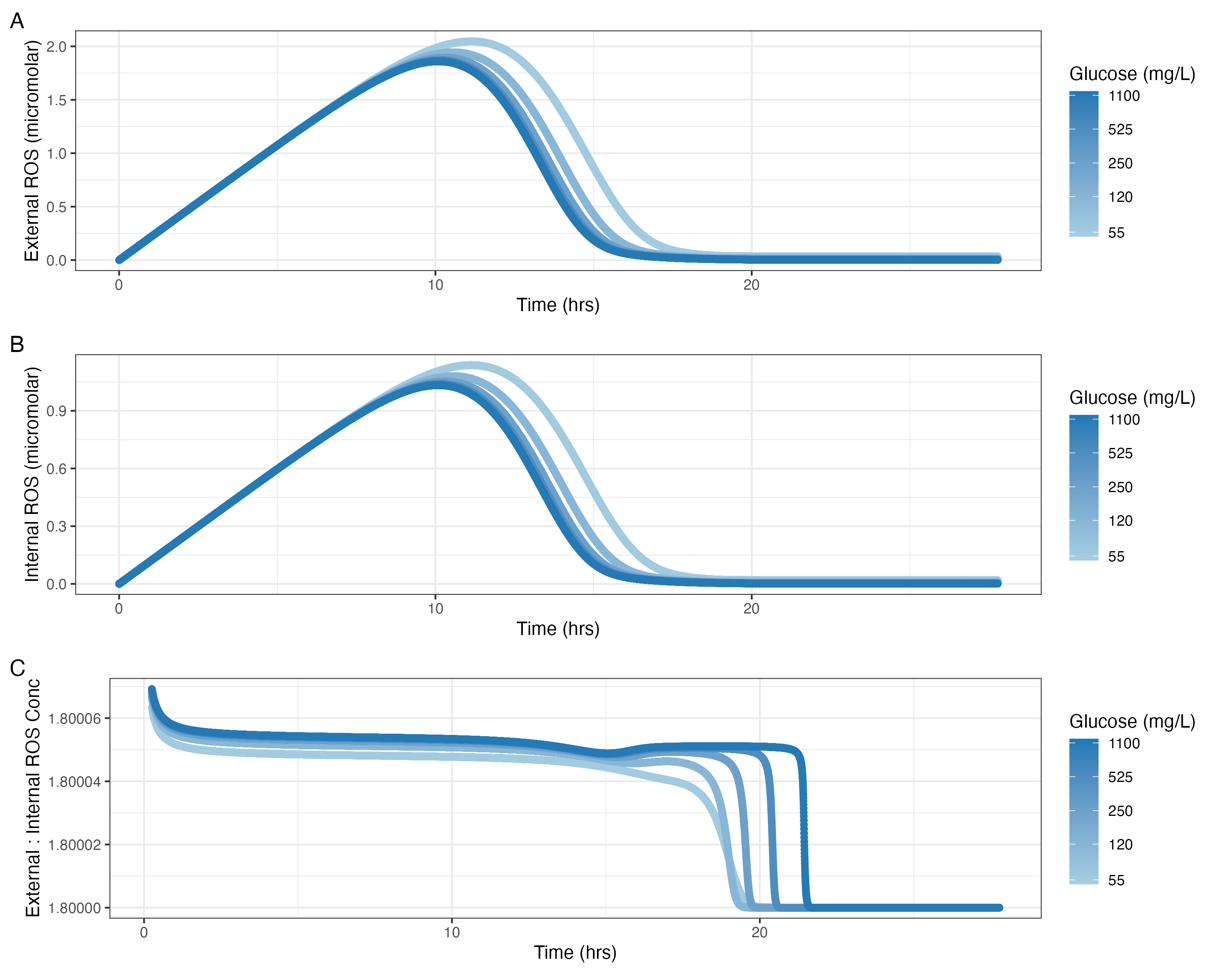


A: External ROS dynamics, B: Internal ROS dynamics, C: External to internal ROS ratio dynamics showing maintenance of ~1.8 fold lower ROS within the cytoplasm compared to the environment. The first 15 minutes are not shown in panel C due to a numerical transient of high values as a result of dividing by numbers close to limit of numerical precision.

# Figure A9: Model E – ROS removal rate (ahp/kat degradation) increases with internal glucose concentration.


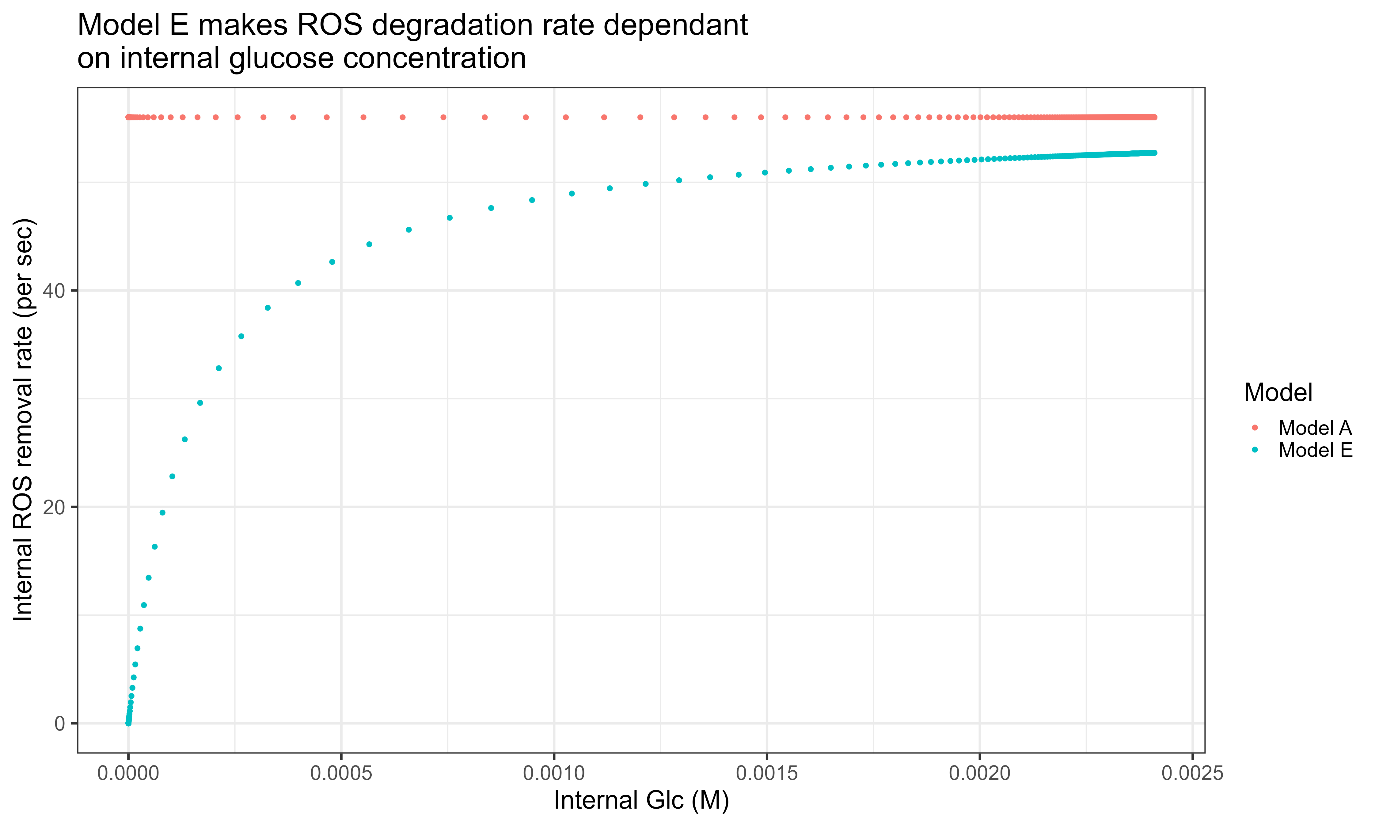


# Figure A10: Model F – ROS removal rate (ahp/kat degradation) increases with cell density


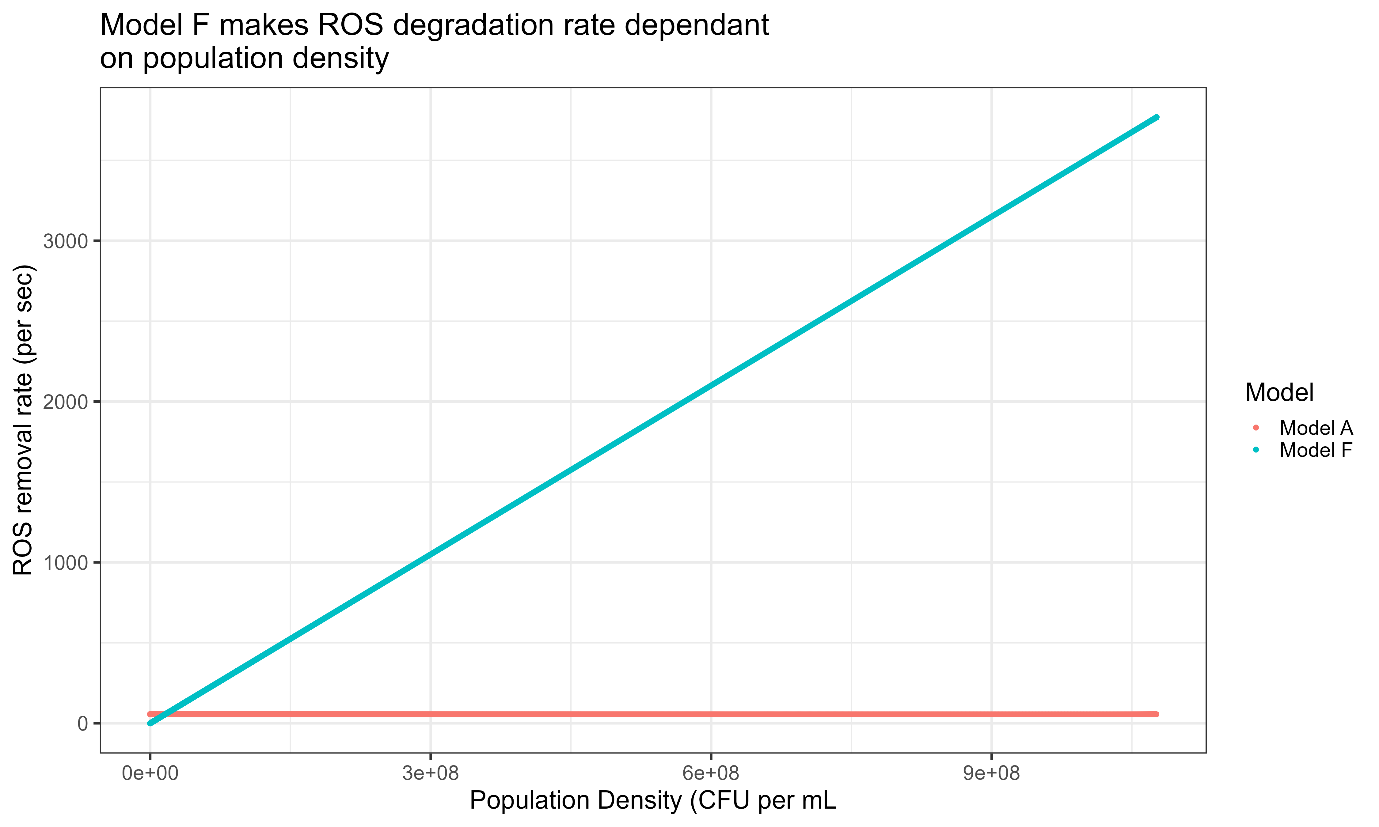


# Figure A11: Model G – odGTP degradation rate (MutT activity) depends on internal glucose concentration


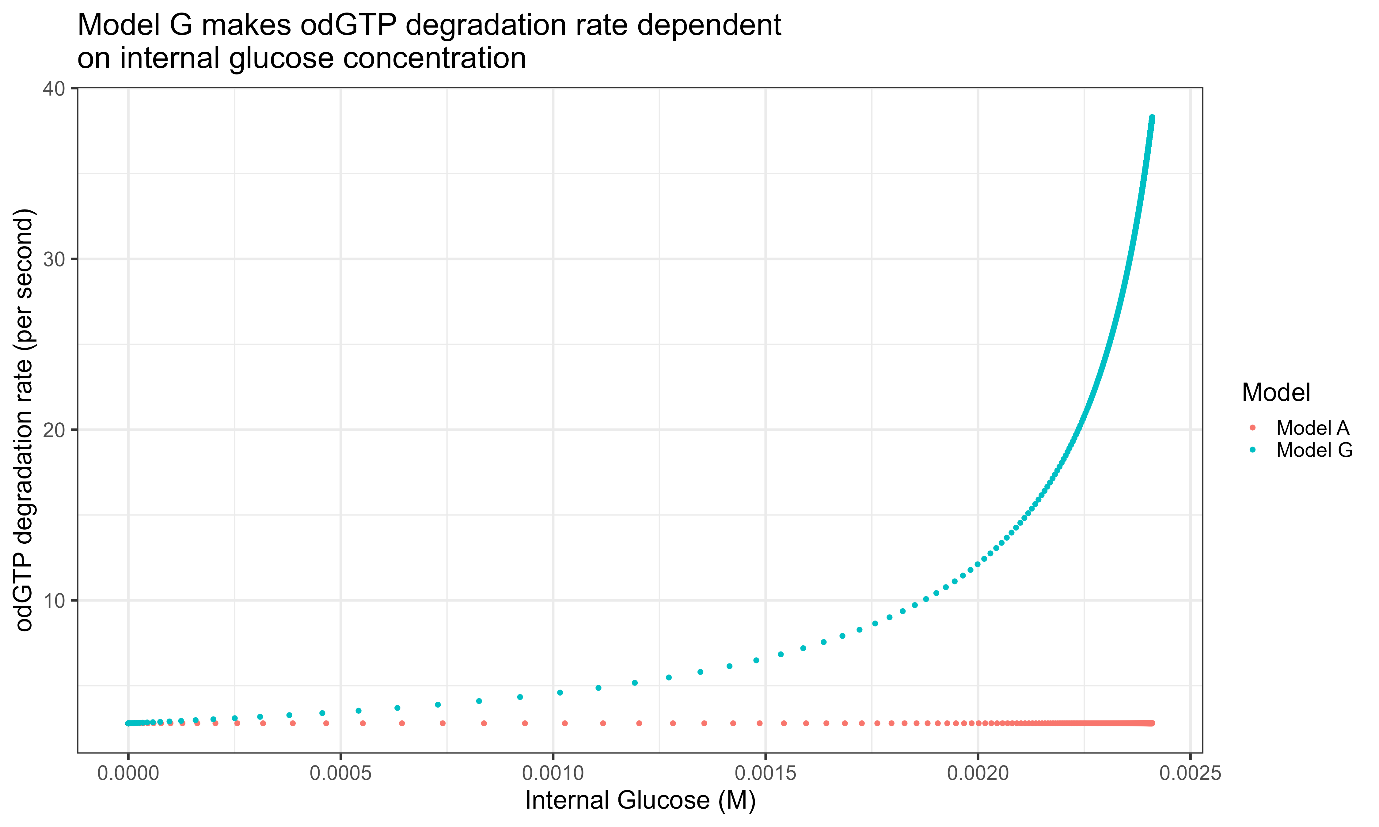


# Figure A12: Model H – odGTP degradation rate (MutT activity) depends on odGTP concentration


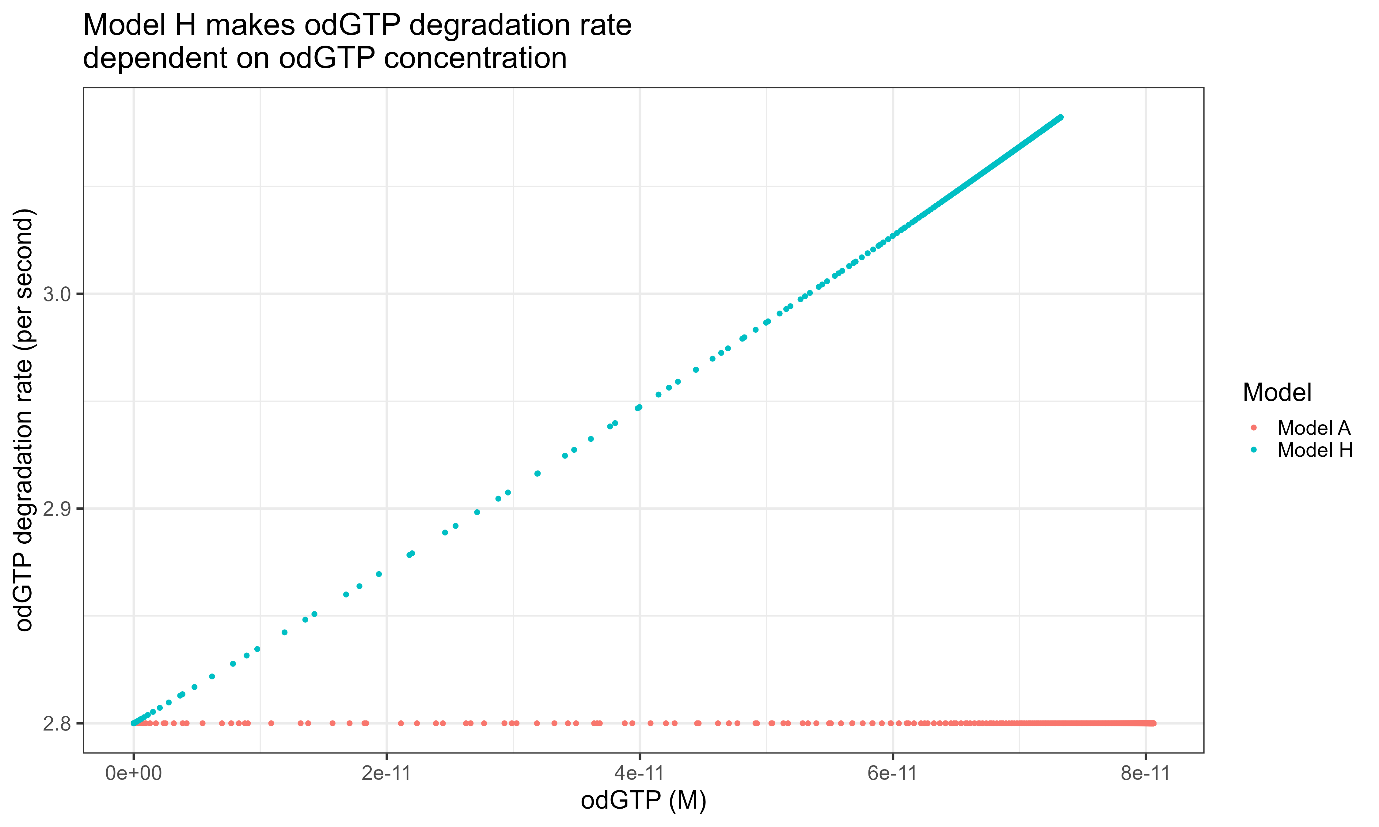


# Figure A13: Model I – odGTP degradation rate (MutT activity) depends on internal ROS concentration


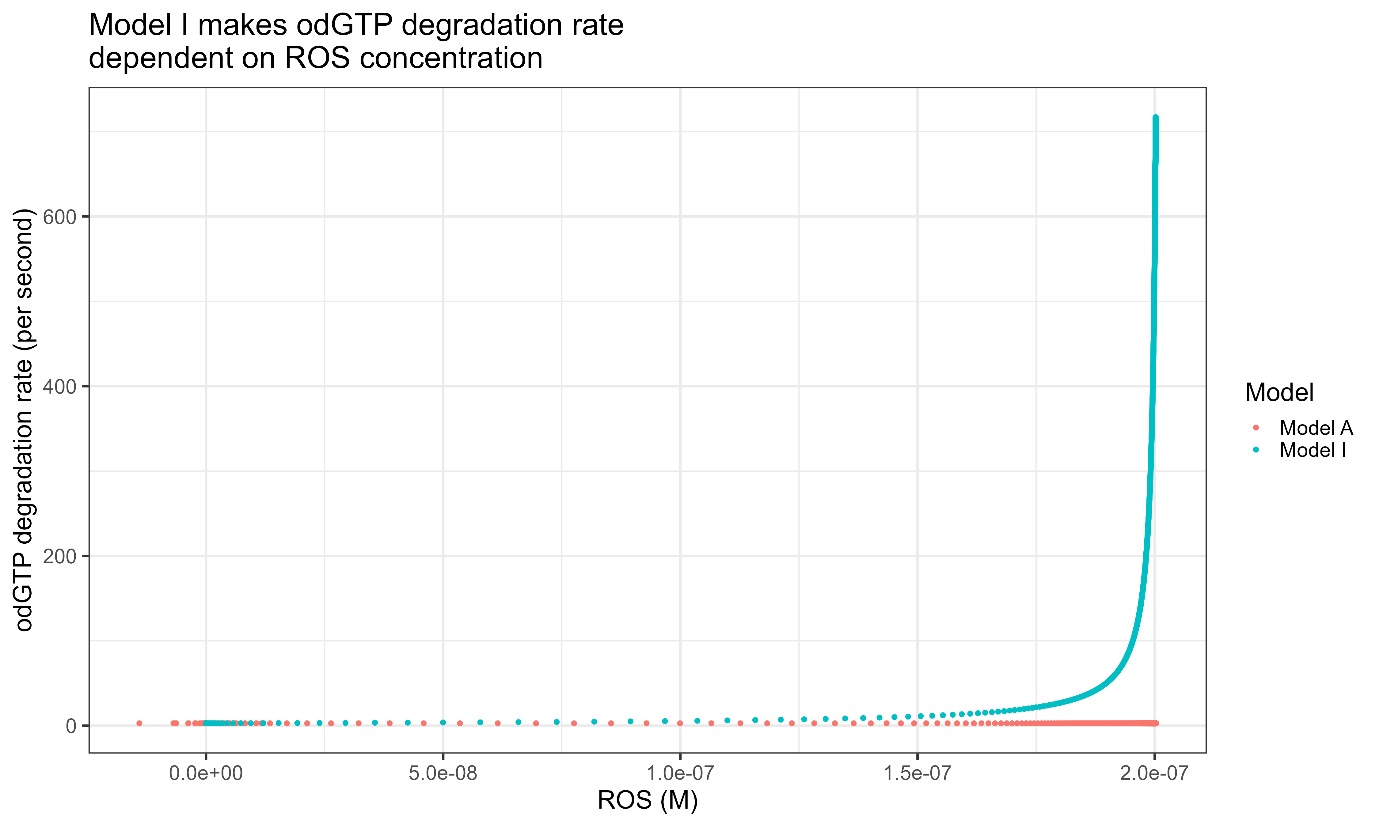


Figure A14: Model J – odGTP degradation rate (MutT activity) shows Michaelis Menten saturating kinetics. As odGTP concentrations remain far below the Km value of 0.48μM the outcome is equivalent to mass action kinetics.


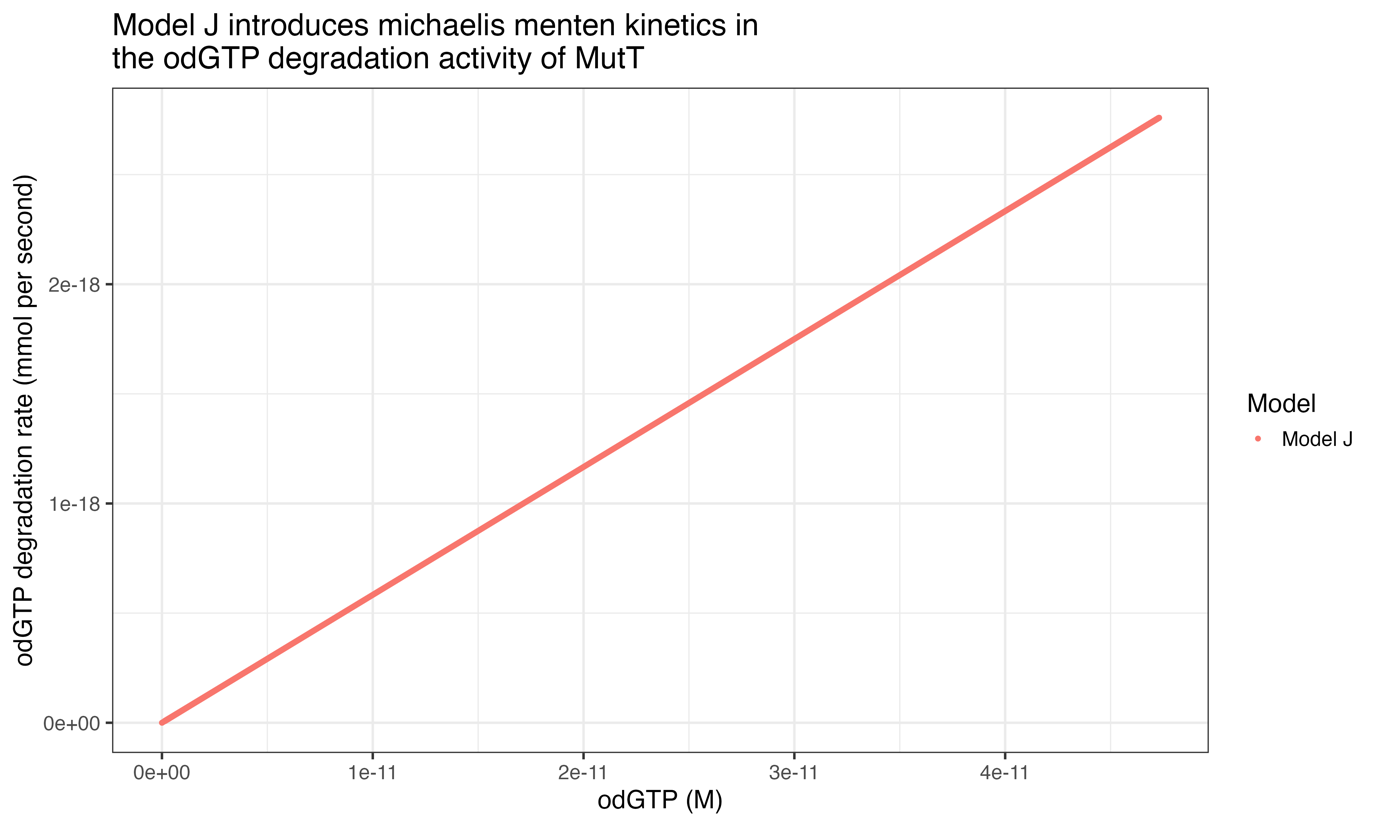


# Figure A15: Model K – Diffusion of ROS across the cell membrane and Michaelis Menten kinetics of AhpCF/KatEG activity are included however no external ROS production is included.


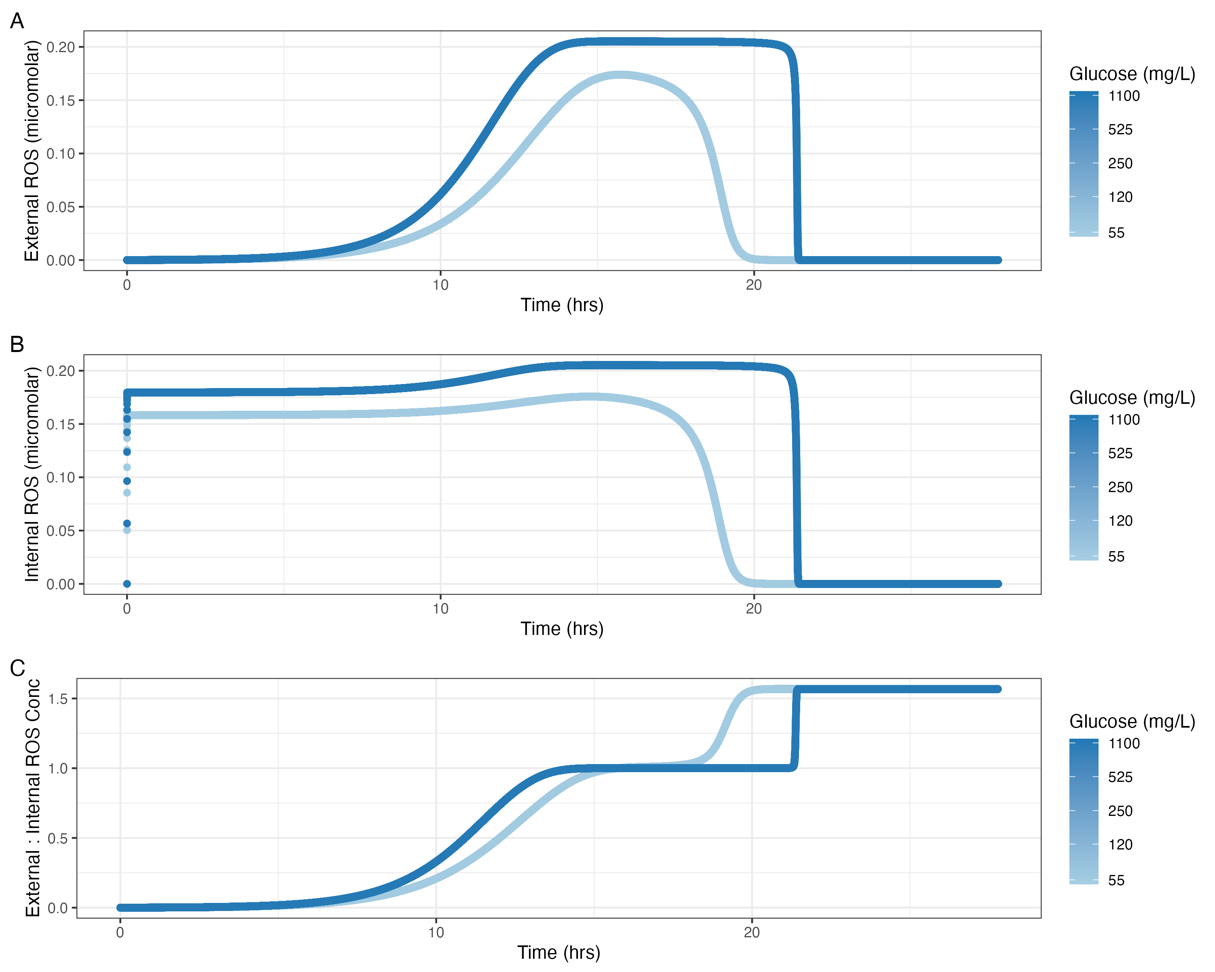


A: External ROS dynamics, B: Internal ROS dynamics, C: External to internal ROS ratio dynamics. Data shown for high density (1100mg/L glucose) and low density (55mg/L glucose) simulations. Without an external source of ROS the concentration gradient across the membrane reaches equilibrium at ~1.5. Simulations were run with time steps of 1 second to give greater resolution of dynamics (as opposed to other runs with 10 second time steps).

1. Uhl L, Dukan S. Hydrogen Peroxide Induced Cell Death: The Major Defences Relative Roles and Consequences in *E. coli*. PLOS ONE. 2016;11(8):e0159706.
