# Supplementary material for: Collective peroxide detoxification determines microbial mutation rate plasticity in E. coli
Source: PLoS Biol. 2024 Jul 15;22(7):e3002711. doi: 10.1371/journal.pbio.3002711 (PMC11272383; doi:10.1371/journal.pbio.3002711)
Supplement: Attachment — Submitted filename: RESPONSE_PBIOLOGY-D-23-02507_R1.pdf [file pbio.3002711.s040.pdf]

REVIEWS:

Reviewer #1: Modelling evolution of biofilm and antibiotic resistance.

Reviewer #2: Bacterial DNA repair and mutagenesis.

Reviewer #3: Christopher Marx. Ecology and evolutionary microbiology.

Reviewer #1: Summary

This Update article extends previous work on density-associated mutation rate plasticity (DAMP) with a computational model to generate hypotheses for the underlying mechanisms. Models that can reproduce DAMP are then explored experimentally to determine the underlying mechanisms of the observation of lower mutation rate in denser populations. Given the fundamental role of mutation rate variation in biology and that DAMP has been observed in taxonomically diverse species this work would be of interest to a wide range of biologists. The authors main conclusion is that the collective peroxide detoxification ability of microbial populations determines mutation rate plasticity. This is based on four main experiments.

A. No DAMP is observed in populations grown under anaerobic conditions
B. No DAMP is observed in E. coli populations deficient in the degradation of hydrogen peroxide
C. No DAMP is observed when the transcriptional regulator Fur is deleted which is expected to lead to higher iron levels in the cell and more ROS damage
D. Reduction of mutation rates in denser populations is restored in peroxide degradation-deficient cells by the presence of wild-type cells in a mixed population

This study is a substantial update to the previous work both in terms of the model and the experimental work. It is not trivial to measure the slopes of mutation rates and the authors have conducted a very large number of fluctuation tests to produce data of high quality. I agree that most of the experimental results are consistent with the authors' interpretation that DAMP is caused by collective peroxide detoxification. However, I do not think that this is the only reasonable interpretation of the experimental results. The authors could do more to discuss and possibly rule out alternative explanations and perform a few additional experiments that could support or argue against their hypothesis. I am overall very positive to this high-quality work, but I cannot currently see that the authors´ conclusions are fully supported by the data presented.

R1.0 Thank you for the positive assessment of our work. We trust that the further experiments and discussion we have now added to the study, outlined below, increase confidence in our conclusions.

Major comments

1. Dissolved oxygen will decrease rapidly as a population grows beyond a certain threshold (about 10^8cells/ml) in a poorly aerated culture such as in deep well plates. I have seen large differences in mutation rates for oxo-G mediated mutations in regular 96-well plates compared to tubes where aeration is better. When oxygen levels are lower then endogenous ROS production will be lower, which could lead to the observed reduction in mutation rates at high cell densities. A simple way to test this would be to see if there is DAMP in well-aerated cultures as well, for example baffled e-flasks with small volume and fast shaking. Under such conditions there should be high dissolved oxygen until at least 4x10^8 cells/ml (https://doi.org/10.1186/1475-2859-5-8). This hypothesis would also be consistent with the main experimental results but would have a different interpretation of the collective detoxification compared to reduced endogenous production of ROS. It could also be formulated as an alternative model where ROS production is decreased when population density increases above a certain level due to lower dissolved oxygen. This model could be experimentally tested as it would predict higher and similar mutation rates at low/medium cell densities as long a dissolved oxygen is high and lower mutation rates due to drastically reduced ROS production at high cell densities.

R1.1 We appreciate your raising the potential confounding factor of relatively poor oxygenation in high density cultures in our experimental setup. We have taken steps to consider the effect this may have and conclude that this does not explain our observations of reduced mutation rates at high population density:

R1.1.1 The reviewer suggests that with different vessels for incubating the parallel cultures we may avoid this deoxygenation effect. In the original work this manuscript updates, we tested the slope of DAMP when parallel cultures of 10mL were incubated in 50mL centrifuge tubes with 250rpm shaking (i.e. with greater potential for oxygenation than a deep well plate). We find that this does not alter the slope of DAMP from the slope we observe under the conditions used in this manuscript (1mL parallel cultures in deep 96-well plates shaken at 250 rpm). See fig.2A in https://doi.org/10.1371/journal.pbio.2002731 , *E. coli* cultures grown in 10mL are plated on nalidixic acid (circles) and in 1mL are plated on rifampicin (triangles). We have now augmented our consideration of oxygenation (see below), including a new figure (S6) which also refers to this previous result in the caption.

R1.1.2 Given that DAMP, as mentioned in R1.1.1, is also observed in cultures grown in 50ml tubes, we measured dissolved oxygen (DO) levels in cultures of high and low density these vessels. These measurements were taken across the growth cycle as DO is shown in the paper suggested by the reviewer (https://doi.org/10.1186/1475-2859-5-8) to vary dynamically over time. As in that paper, we observe oxygen dynamics across the culture cycle (see figure below). However, we also observe oxygenation to be approximately equal among nutrient treatments (red indicating low nutrients giving low population density). These results are now included as Fig. S6 (shown below) and discussed on L294-302.

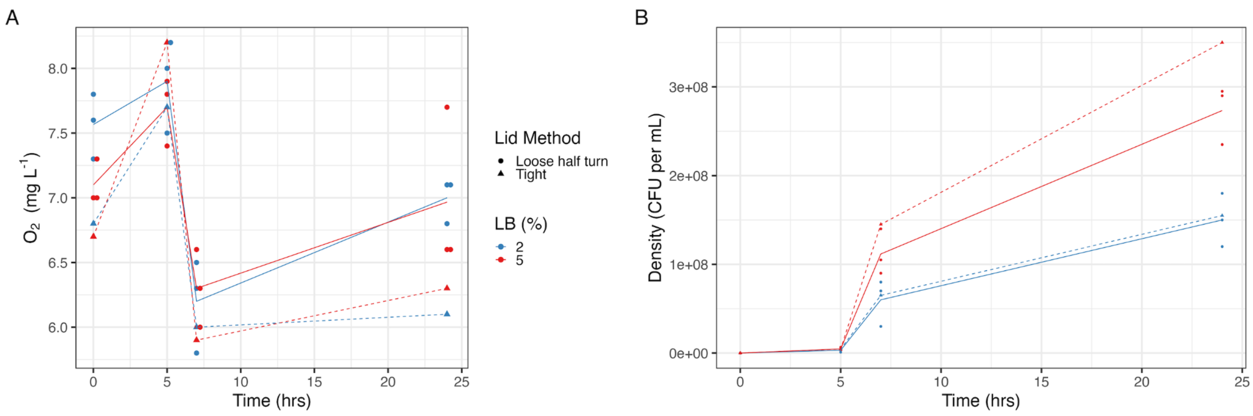

R1.1.3 If DAMP were simply the result of poorly oxygenated cultures at higher density (the reviewer suggests above 'about 10^8 cells/ml', which seems an appropriate threshold to us too) then one might expect little or no density association to be observed below such high densities, certainly we would expect the association to be different. We find the slope of DAMP not to be significantly different ($t=1.25$, $DF=457$, $P=0.21$) below or above a density of 1e8 cells/mL. Indeed, the best estimate of the slope is actually steeper at low density: at low density (Density < 1e8) slope = -0.95±0.33 (N = 11 fluctuation assays, 95% CI) whilst at high density (Density > 1e8) slope = -0.71±0.17 (N = 59 fluctuation assays, 95% CI). Now reported at L302-309.

2. Fig S4 shows that the slopes were measured at quite different cell densities for different strains/conditions, which is problematic when they are compared only in terms of slopes in the main figures. For example, the catalase and additional of H2O2 experiments are done with populations of high density and the Hpx- mutant has much lower cell density when calculating the slope, presumably because of a growth defect. Obviously, this would be problematic if ROS degradation is directly related to cell density but also if the dissolved oxygen level is important for mutation rates.

R1.2.1 As shown in Fig. S5 (and more explicitly tested in R1.1.3 above), we find the logarithmic response in mutation rate to be remarkably linear in response to log density (as manipulated by nutrients). This appears to be the case for all the strain environment combinations we have tested and primarily breaks down only at the highest population densities in rich media, where stress induced mutagenesis is activated (https://doi.org/cst8 now reference 14), which we do not focus on here. This gives us some confidence in comparing slopes among strains/environments where the population densities only partially overlap.

R2.2.2 Nonetheless, we agree that confidence in slope comparisons will be greatest when they are made among populations at similar density and indeed similar average mutation rates. While, ultimately, this is not fully possible (for instance through pleiotropic growth defects as the reviewer highlights), we have now conducted further mutation rate estimates, with a view to extending the density ranges over which our mutation rate slopes are estimated. We have thus conducted ≥4 further fluctuation assays for each of the highlighted treatments with limited data: low density + catalase, low density + additional H_2O_2 and high density Hpx^-. These are now included in all relevant data analysis, in figure 3 and in supplementary figures S1,5,8,9,10,11,16,17 & 18. New data points are highlighted in black in this modified version of Fig.S5 for the reviewers:

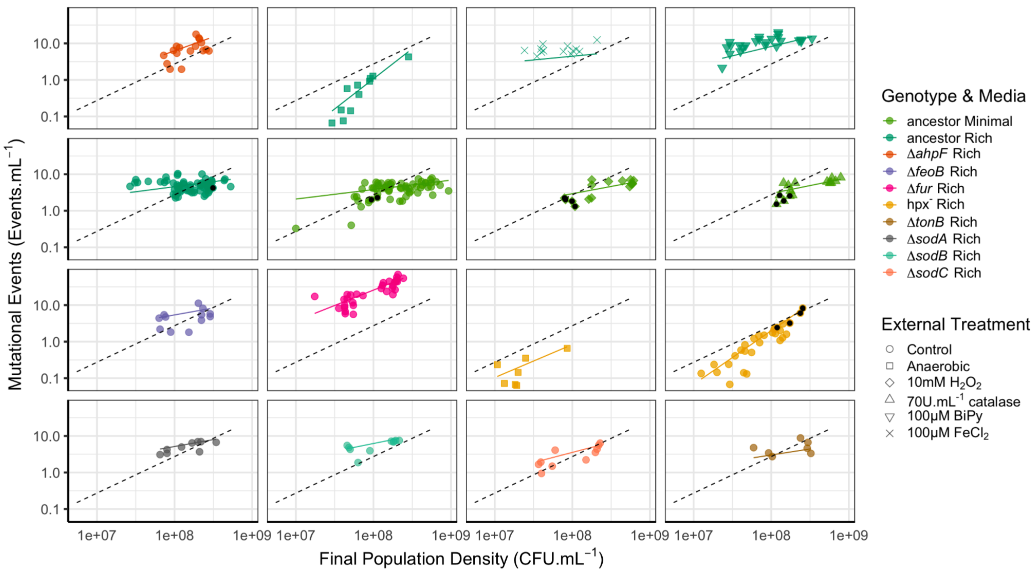

3. A related point is that cell density here is determined by nutrient amount. This would also change other experimental conditions, for example how long a population has been in stationary phase. Would DAMP also be observed if mutation rate was measured at different time points during growth in the same concentration of nutrient? If this has already been confirmed in a previous study, it would be good to mention as it would strengthen the hypothesis that is cell density and not another correlated factor that is the key determinant of DAMP.

R1.3.1 As you suggest, disentangling correlated effects such as growth rate, nutrient provision and population density is an important part of understanding DAMP. We have previously shown all three of these factors to have separable effects on mutation rates, this work is not yet published but we have now included a citation of the PhD thesis in which this work can be found along with discussion of these potential confounding factors (https://research.manchester.ac.uk/en/studentTheses/ecological-effects-on-mutation-rate, Chapter 4). We also now make this point more explicitly in the methods at line 1591.

In other work cited on lines 842 and 1125 we have shown the slope of DAMP not to differ across the culture cycle https://www.research.manchester.ac.uk/portal/en/theses/evolution-of-evolvability-understanding-mutation-rate-plasticity(2d8e5e78-dcd7-4d8c-b37e-07da62592644).html.

R1.3.2 We have also now simulated the slope of DAMP in the dynamic model in both exponential and stationary phase (Fig. A4 in Appendix 1), this shows the slope of DAMP not to substantially differ based on growth phase in either the baseline model or the most important model variants (D and F).

4. I find it problematic that the Hpx- strain has secondary mutations including a fecD deletion that is likely to have a major impact on mutation rate results as it will probably have lower iron levels in the cell and a lower mutation rate with could contribute to loss of DAMP. I realize that it would be a substantial effort to construct a new Hpx- mutant and that it is possible that a new mutant would also have secondary mutations. However, confirming key results with an independently constructed Hpx-

strain would substantially strengthen the article and perhaps it would be possible to obtain a previously constructed strain for example that used in https://doi.org/10.1007/s00253-021-11169-2 or the ΔahpCF katG mutant used in (https://doi.org/10.1046/j.1365-2958.2001.02303.x). It might also be possible to repair the fecD gene or complement it by addition of a copy elsewhere in the genome. The conclusions rely heavily on this one strain and fecD and the other mutations makes
it uncertain if the phenotypic effects are only due to the deletions of ahpAC, katE and katG.

R1.4.1 As suggested by the reviewer, we have sourced an independent Hpx^- strain from the lab of Benjamin Ezraty (colleague of Patrice Moreau https://lcb.cnrs.fr/team/ezraty/). We find DAMP to be absent in this strain (X^2=0.808, DF=1, P=0.369), this result is now included as Fig.S8 and discussed on L383-386.

5. In the computational model ROS is the only source of mutations. In model D the model is changed to constant ROS production. I find it very problematic that this ROS production is then simply divided between the cytoplasms of all cells (line 593). This will obviously lead to extremely high levels when there are few cells and low levels when there are many cells which explain why there is DAMP, but it is not realistic that all ROS produced in the environment is suddenly allocated to the inside of the cells present in the population.

R1.5.1 We are also troubled by the lack of realism in how external ROS production was transferred into internal ROS by model D. We have therefore now modified model D (external ROS sources) to include features of model K (explicit diffusion of H_2O_2 across the membrane). This modified version of model D is now included in the manuscript simply as 'model D'. This change of model does not substantially change the DAMP slope (Model D (old) slope = -0.9131 , Model D (modified) slope = -0.906).

- Interestingly, in this new model, lower permeability of the membrane leads to decreased DAMP and decreased mutation rates. This is now discussed in L214 – 216 and shown below (Figure A5 in Appendix 1).

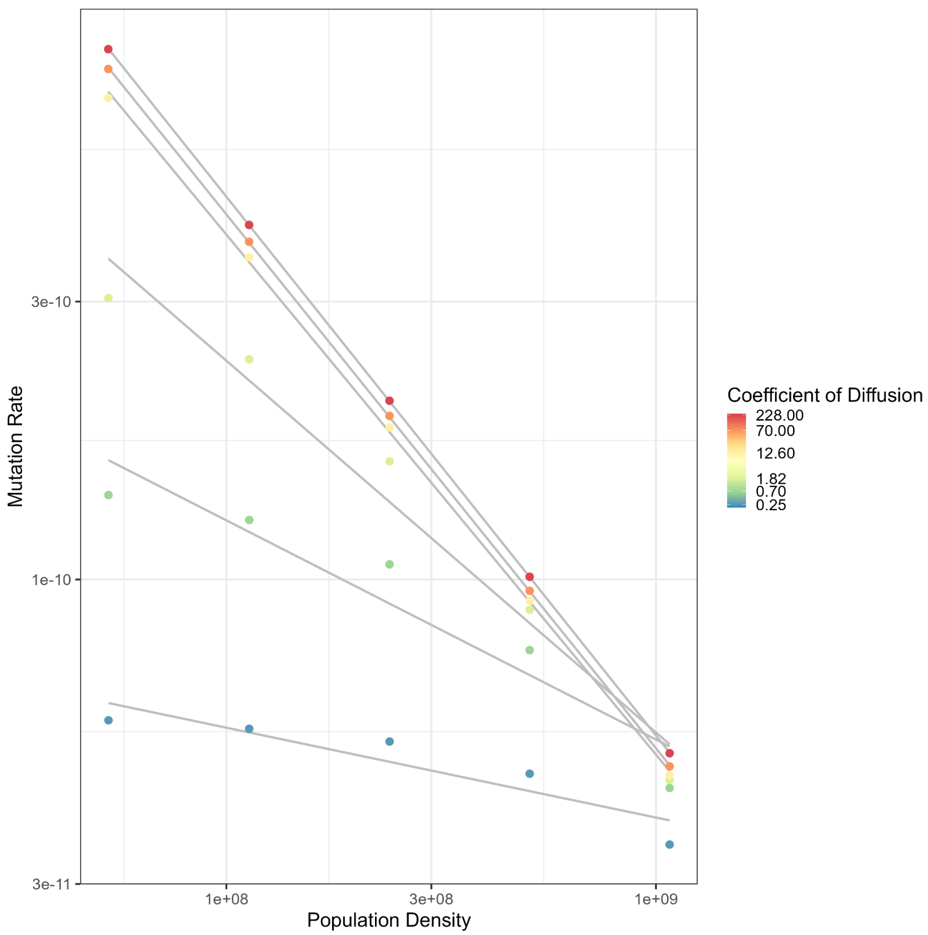

R1.5.2 We have also now included ODE modelling of the coculture experiments showing the predictions of our updated model D to be in good agreement with lab data (see plot below). This is now included as Fig. S11 and discussed on L405-407 and L802-868.

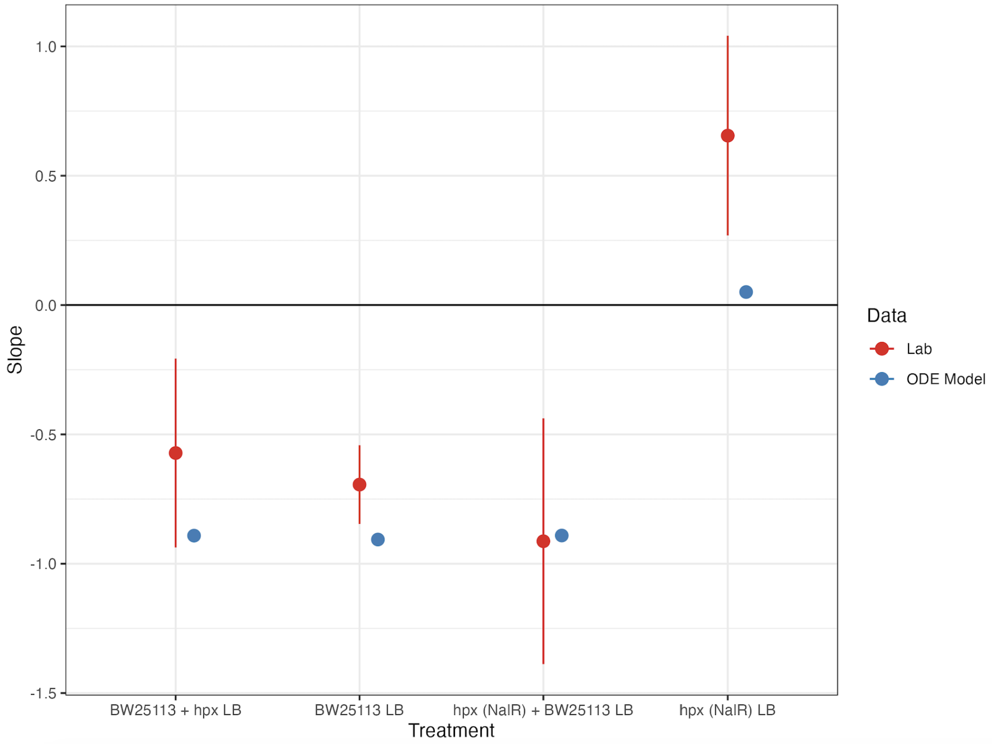

(95% CI for the ODE model estimates are narrower than the points and so are not visible.)

6. In Model F, the other model showing DAMP, ROS degradation is directly determined by population density and as ROS is the only source of mutation this will obviously lead to a higher mutation rate at lower densities. Thus, this result is self-evident

R1.6.1 All 10 model variants are constructed to produce scenarios that one would expect to produce DAMP. Despite this only 2 of the 10 variants produce a stable DAMP phenotype. This is because of the effect of feedbacks within the model and makes testing models structures which seem self-evident, worthwhile. We have now made this logic clearer at L178-183.

and there is no attempt to test this model experimentally for example by measuring expression of ahpCF, katE or katG at different cell densities.

R1.6.2 It would be very interesting to measure expression of these genes across a density gradient in future work. However, the important finding here is that DAMP can be reproduced either by altering Kat/Ahp expression in response to density (model F) or by introducing external ROS production (model D). The contrast is that without exogenous ROS production (model F) mutation rates depend on the cytoplasmic concentration of Ahp/Kat (expression level) whilst when ROS production is primarily exogenous mutation rates depend on the total number of Ahp/Kat enzymes in the system (cell density). This is now discussed at L349-352.

7. Line 297-300. Adding environmental H_2O_2 or catalase do not disrupt the negative relationship between mutation rate and population density. I think this result needs to be discussed more and

explained how it is consistent with the authors' hypothesis of how DAMP works. To me the interpretation would be that environmental H2O2 is not important.

R1.7 This is an important point and we have now included further discussion of it on L333-335 where we cite the paper ‘Alkyl Hydroperoxide Reductase Is the Primary Scavenger of Endogenous Hydrogen Peroxide in *Escherichia coli*.’ 10.1128/JB.183.24.7173-7181.2001.

Given the enzyme kinetics of catalase and alkyl hydroperoxidase it is AhpCF which will have a greater protective effect against mutations than catalase in the absence of additional H_2O_2 (see plot below). Thus we reason that the limited effect of added catalase indicates that the high levels of peroxide, with which catalase can engage, are not important to DAMP, rather it is the low levels of peroxide dealt with by Ahp that matter, which is also why we focus on the *ahpF* deletant.

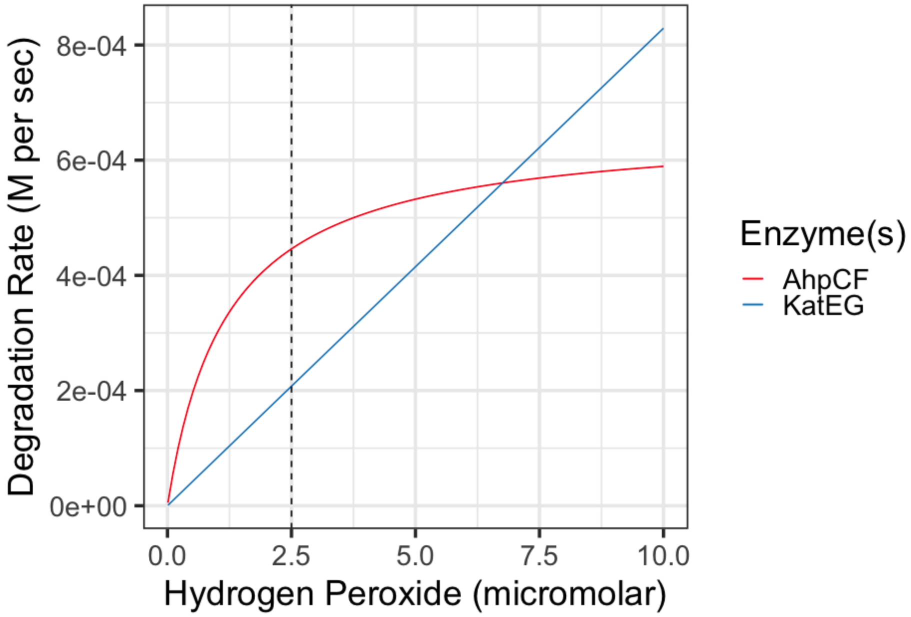

Enzyme kinetic plot for AhpCF & KatEG given k^{cat}_{Ahp}[Ahp] = 6.6e-4 M/L/sec, KM_{Ahp} = 1.2e-6 M, k^{cat}_{Kat}[Kat] = 4.9e-1 M/L/sec and KM_{Kat} = 5.9e-3 M (Uhl & Dukan, 2016, PLoS One) we calculate degradation of ROS by AhpCF to proceed at a rate 2.15 fold higher than KatE/KatG (4.5e-4M/sec compared to 2.1e-5M/sec) (this concentration is indicated by the dashed line in the plot below).

Minor comments

8. In the Variable table (line 493) WtCell and mCell is listed but in later equations the variables are named differently as dGcell (line 527) and dmGcell (line 537).

R1.8 This inconsistency has been corrected.

9. Ref 15 (line 78) reports increased AT->GC transitions in low density populations. Yet ROS associated mutagenesis through 8-oxo-G is known to give rise mainly to transversions with an increase

in A->C transversions in a mutT mutant and increased G->T transversions in mutM mutY mutants. Sanger sequencing of rpoB for a selection of rifR mutants could be done to confirm that ROS associated mutations are more common at lower cell densities, which is fundamental to all models proposed here.

R1.9 This is discussed as an open question on L494-503 since it is beyond the scope of this manuscript. Nonetheless the preprint cited (ref. 15) is ours (currently in press at *Microbiology*), which does indeed open up the possibility of different plasticity mechanisms going on with different members of the mutational spectrum. The modelling here could certainly be expanded in future to be explicit about this spectrum (e.g. distinguishing A->C and G->T transversions in GO-related pathways and expanding to transitions and MMR-related pathways). However, none of this invalidates the current work, where the mechanism identified (collective environmental detoxification leading to reduced mutation rate at high population densities) may or may not be specific to the single mutational mechanism on which we built the model, a point we now make explicitly at L494-503.

10. In lines 325-326 fur is described as the master regulator of intracellular iron. It would be helpful for the reader to know that Fur not only regulates genes related to intracellular iron but that it is a major regulator of >100 genes, which also included gene with other roles (https://doi.org/10.1038/ncomms5910) including ROS defense genes like katE and katG (https://regulondb.ccg.unam.mx/regulon/RDBECOLITFC00093).

R1.9 We have extended the explanation of the role of Fur on L368-369 to highlight the regulation of gene classes beyond iron import such as *katE/G* and *sodB/C*, including citing the Seo et al. reference highlighted.

11. Fig S6 shows relatively small differences in hydrogen peroxide concentration between low and high nutrient levels. Would this be enough to explain the difference in mutation rate?

R1.11 This is an important point. While small relative to experimentally imposed peroxide stress, these differences are large relative to typical amounts of environmental peroxide (>2-fold). We can get at an answer to the question by asking whether the scales of change in peroxide and mutation rate are similar. We originally analysed the relationship between cell density and environmental H_2O_2 on a linear scale whilst DAMP is measured as a log-log relationship, making this comparison difficult. We have now re-analysed the relationship between environmental H_2O_2 and population density (Fig.S7A) as a log-log relationship; this makes the results directly comparable to the DAMP phenotype. Under this new analysis the log-log relationship between population density and environmental H_2O_2 is -0.33 ± 0.1 (95% CI) in both rich and minimal media. This is very much in line with our measurements of DAMP from the same strain (wildtype MG1655) where the log-log relationship between density and mutation rates is -0.43 ± 0.25 (rich media, 95% CI) or -0.58 ± 0.24 (minimal media, 95% CI), as given in table S1. This has now been clarified in the text (L478-481) and Fig.S7A updated to be plotted on a log scale. The plot below for the reviewers combines mutation rates and H_2O_2 in one plot to make this comparison more visually clear.

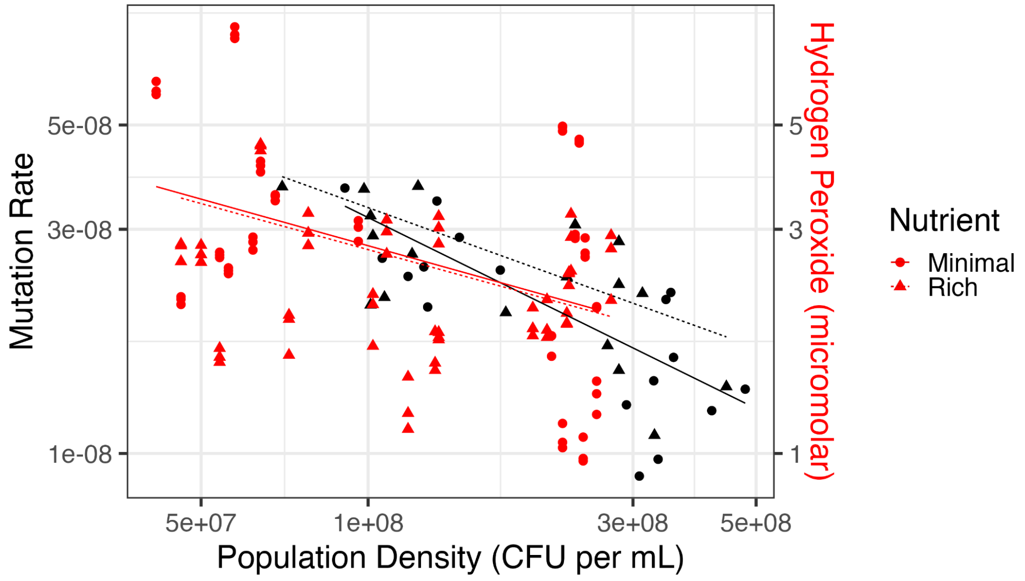

Reviewer #2: The manuscript by Green et al. examines the mechanisms underlying the phenomenon that cellular mutation rates decrease as the density of cells in culture increases (DAMP = density associated mutation rate plasticity). This effect has previously been discovered and described by the labs that performed this study. The present manuscript goes a step further in testing specifically the hypothesis that the differences in mutation rates can be explained by the capacity of a culture for scavenging mutagenic reactive oxygen species (ROS, specifically H2O2). The authors developed a phenomenological model of ROS-induced mutagenesis dependent on the concentration of glucose in the culture media. Here, glucose concentration affected the rate of H2O2 production and the population density. This alone was insufficient to explain the DAMP effect, so the authors systematically varied parameters and model structure to recapitulate the effect. According to the story in the manuscript, this led to the realisation that ROS scavenging capacity of the culture varies with density, which in turn affects mutation rates. This was then tested via a series of mutation rate measurements with E. coli strain deficient in H2O2 detoxification mechanisms and iron regulation. While H2O2 scavenging-deficient cells showed no DAMP effect, a co-culture with wild-type cells restored the effect, showing that it is indeed a collective phenomenon. Overall, the article is interesting and provides clear insights into a complex phenomenon.

R2.0 Thank you for the positive assessment of our work.

I suggest addressing the following minor points:

1) Abstract: "in vivo mutation rate estimation". The phrase "in vivo" could be misleading as it might suggest mutation rate estimation of bacteria within hosts or in the environment, as opposed to "in vitro" lab culture conditions.

R2.1 All references to bacterial cultures have been changed to ‘in culture’ rather than in vivo or in vitro to avoid confusion.

2) Introduction: "We show that this density effect is also experienced by cells deficient in H2O2 degradation when cocultured with wild-type cells able to detoxify the environment." Authors should cite here the classic Ma & Eaton 1992 paper showing that cell survival of a scavenging mutant strain is rescued by addition of wild-type cells. The paper is cited in the discussion section, but should be acknowledged more prominently in the introduction & results. Specifically, in line 246: "We would further expect dense populations to show greater removal of environmental ROS than low-density populations." This was shown by Ma & Eaton, and should therefore be cited as consistent with the model prediction, rather than presented as a new hypothesis that was generated by the model here.

R2.2 We have now cited this paper more prominently in the introduction, clarifying in L96-99 that the novel element of our hypothesis is not the existence of group protection from H_2O_2 but the relevance of this to mutation rates and without additional H_2O_2; in contrast the Ma & Eaton paper which explores group protection against extreme conditions of 1mM H_2O_2 (~500 fold higher than concentrations measured in our media).

3) Line 240: "Model F, which gains DAMP relative to model A, describes an increased rate of ROS detoxification dependent on the population density. This reflects a system in which ROS detoxification is primarily occurring within cells." Model F is described in lines 612f. It was unclear to me why the model does not include a scavenging enzyme activity to remove ROS?

R2.3.1 The rate of ROS detoxification by scavenging enzymes Ahp/Kat is given by parameter O3, in model F this is replaced by 'C3a x Cell Density'. As stated, this makes the rate of ROS detoxification (by scavenging enzymes) dependent on Cell Density. We have clarified this in the methods on L704-706 and in table 2.

Why does the removal rate depend on the parameter GCperGen?

R2.3.2 wtCell is a measure of the molar concentration GC pairs in genomes. How that molar concentration relates to cytoplasmic volume (eq. 6) and population density and hence peroxide removal rate in model F (eq. 7_f) therefore depends on the number of such GC pairs per genome (GCperGen). Thus, wtCell is converted to a cell density in units of cells per mL by multiplication by molML (avogadros number / 1000, particles in 1ml of 1M solution) and division by the number of GC pairs in the genome of MG1655 (GCperGen). Therefore, the expression 'molmL/GCperGen * wtCell' calculates the population density. This is now clarified in line 704-706.

4) In general, I found it a bit difficult to follow the description of the various model modifications. The text often refers to specific changes in equations, but these aren't shown in the main text. The figures are also quite minimal (which is good), but I think there could be an argument for including a schematic that illustrates the changes introduced by the models A to K.

R2.4.1 We have now included flow diagrams of the changes introduced by all 10 model variants as Figure A1 in the newly added Appendix 1 to help readers follow this section.

R2.4.2 In this process we also discovered an error in the parameter C3c which has now been slightly altered from 1.98e-7 to 2.01e-7 with no resulting effects on our conclusions.

5) Line 369: "Some difference in plating efficiency between the two hpxnalR&rifR strains was observed (Fig. S9), this is likely due to the pleiotropic effects of RifR resistance mutations in the rpoB gene (42, 43)." Do the two hpxnalR&rifR isolates have different RifR mutations?

R2.5 We have now sequenced the *rpoB* genes of the two $Hpx^{-}_{rifR\ nalR}$ strains used in the reconstruction test and find their resistance mutations to be at different sites leading to changes at different amino acid residues. These results are now included in Fig. S12 and the sanger sequencing method has been added to the methods section. Such different mutations are indeed likely to have different pleiotropic effects on growth, which we can and do account for when looking across many mutations in our estimates of mutation rate (now Fig. S16) but are less readily controlled for when focusing on specific strains, as here. We now make this explicit in the text at L424-426.

Reviewer #3: This paper takes on a really remarkable phenomenon - the nearly linear increase in mutation rates with decreases in population density (DAMP). I only first learned about this phenomenon earlier this year and find it fascinating, both in terms of the physiology and in the implications. As microbiologists, we tend to think of microbes as blind, independent replicators oblivious to other microbes in their midst other than metabolic use/cross-feeding or production of particular toxin molecules. As evolutionary biologists, we think that changing cell number (usually via density) only has demographic effect in terms of drift and mutational supply (which would scale linearly with Ne). If DAMP nearly erases the effect of using density to change demographics, then how should we interpret our experiments?

In light of this, I really appreciate the approach taken here to attempt to generate a quantitative model of these processes and see what parameters or structural variants are consistent with the empirical findings, then follow up with further experiments to target those processes. I think the clarity of the description of the model and the step-by-step move through models that have no DAMP (or reverse DAMP) is commendable. This lands upon two scenarios that would involve ROS mainly arising from the medium itself and that ROS degradation is key to removing this pre-existing ROS in a density-dependent manner. I think the results are compelling.

R3.0.0 Thank you for the positive assessment of our work.

As photochemistry is blamed as the main source in media (line 197), this leads to an alternative experiment where one attempts to change the level of ROS in the medium prior to the experiment to see if that would simply result in "high-density" mutation rates even at low density.

R3.0.1 This would be an interesting experiment and asks a similar question to our fluctuation assays in the presence of catalase (see R1.7 for discussion). However, the difficulty with the experiment you describe is that with the small innocula required for a fluctuation assay, significant growth is not seen until ~14 hours (glucose minimal) or ~5 hours (dilute LB). By this point the H_2O_2 levels are likely to have substantially increased (see Fig. 6A/B in https://doi.org/10.1016/j.freeradbiomed.2018.03.025). This combination of fast H_2O_2 formation and slow entry to the exponential growth phase make removal of initial peroxide likely to be ineffective at changing the conditions experienced by cells in exponential growth. Therefore, rather than this suggested experiment we have augmented our dataset on mutation rates in the presence of catalase (see R2.2.2).

The key experiment for me was the co-culture of hpx+ and hpx- cells together; that really seems to nail the point. It also argues that the suggestion (line 410) of mixed species communities collectively reducing ROS is a key point.

I do not have any large critiques, but I do have some minor suggestions:

- Figure 1 - I like seeing all the parts and the dynamics. But this was for the base model that -doesn't- show DAMP. And it doesn't show ROS levels through time!!! I think you need to show ROS.

R3.1.1 We have now included the dynamics of ROS in figure 1 (alongside the glucose, density and mutation rate).

And if possible, maybe that and a version of panel D for a version of the model that does show DAMP.

R3.1.2 We have now included the dynamics of each model's mutation rate over time as figure A3 in Appendix 1 (plot included below), the appendix also shows ROS dynamics for model D which shows DAMP (figure A8 in Appendix 1).

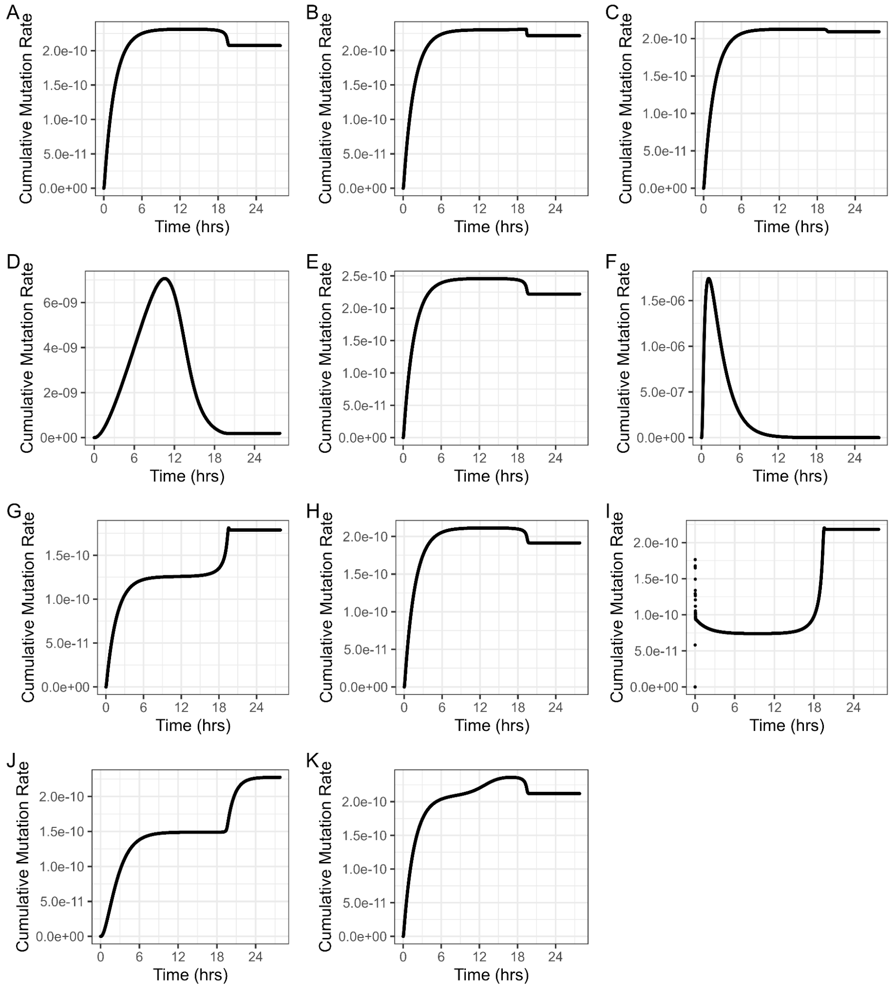

- Figure 1 - It was also unclear why there was a mutation rate during stationary phase. Is that how one should interpret the lines in panel D at 24 hours?

R3.2 The mutation rate shown in figure 1D (now 1E) is not a transient mutation rate but a cumulative mutation rate measured as the number of mutational events divided by the number of cell divisions, in the same way that the laboratory measurements in the fluctuation assays are cumulative over 24h. Thus, the stationary phase culture does have a 'mutation rate' by this metric but once the cells enter the stationary phase this does not change over time.

Please note: for consistency we have now updated Fig.1E (mutation rate dynamics) to calculate mutation rate as mGcell/(mGcell+wtCell), previously mutation rate in Fig. 1E was calculated as mutant cells/total cells. The values of mGcell and wtCell (molar concentrations of mutant and wt GC base pairs) differ only from the total numbers of mutant and wildtype cells by a constant multiplication factor. This change therefore leaves the mutation rate values unchanged aside from very early in the growth cycle (<~5hrs) where the value calculated is influenced by the small value of the denominator rather than the ratio of interest. These inconsistencies at very small values are of mathematical rather than biological origin and are not important to the conclusions drawn as we only measure the mutation rates of these simulations at >24hrs (or 16 hrs in the case of Fig. A4 in Appendix 1) where either method will give identical results.

COMMENTS FROM THE ACADEMIC EDITOR:

Overall, the model structure and formulation is poorly explained and justified, and so several aspects of the model are either unclear or puzzling.

E1.1 We have made changes to address all points raised above as at all unclear or puzzling in understanding the models (R1.5.1, R1.5.2, R1.6.1, R2.3.1, R12.3.2, R2.4.1, R3.1.1, R3.1.2). In doing so we have now included an appendix with various illustrations on ODE model dynamics to improve their clarity to the reader. We would at the same time highlight the comment of Reviewer 3 that "the clarity of the description of the model … is commendable".

For instance, Line 118: why is the mutation rate calculated as the ratio of mutated versus unmutated bases? It should be mutated versus total bases.

E1.2 This is true in principle however, in practice these ratios are essentially equal due to the number of mutated bases being ~2e10 fold smaller than the number of unmutated bases. We have nonetheless now updated all mutation rate estimates to be calculated as the ratio of mutated versus total bases and clarified this in the model description on L561-562 (Fig.1 caption).

From table 1, the units for mutation rate don't add up to per base per generation (in particular, I am not sure how the "per gen" part comes in).

E1.3 This is, at first glance, confusing, however the ratio of mCell:(wtCell+mCell) does give us the mutation rate in units of per bp per generation. This is because wtCell+mCell = GCperGen x total cell count. Therefore we are dividing the number of mutational events (mCell) by GCperGen (the target size of total base pairs that could be mutated) and also by the total cell count (resulting in the per generation part).

$$\frac{mutant\ GC\ base\ pairs}{total\ GC\ base\ pairs} = \frac{mutant\ GC\ base\ pairs}{GC\ base\ pairs\ per\ cell\ \times\ total\ cells}$$

$$Units: mutations \div (mutational\ target\ size\ \times generations)$$

$$= m\ \ bp^{-1}gen^{-1}$$

Although mCell and wtCell are measured as molar concentrations, not counts of base pairs, due to the fixed 1mL volume of the system the units of volume cancel out in the division.

One of the parameters (rate of dGTP oxidation, line 124) is fixed so as to obtain the observed mutation rate at a fixed population density. Presumably, this means that this parameter cannot vary as a function of density, which is an important assumption that cannot be tested.

E1.4 It would be possible to vary this parameter (O2) with cell density as we have done with other parameters. (e.g. simply fit O2 to give the expected mutation rate at our central density (250mg glucose per L) and then allow it to vary up and down at higher and lower densities). However, we chose to focus on the particular model variants we did for the reasons set out in detail on L166-183.

I am also unclear why the parameter r is a relative rate (ROS production relative to dGTP production).

E1.5 This ties cellular ROS production to the metabolic rate / growth rate using dGTP production as a proxy for this rate. We have relaxed this assumption in models C & D where ROS production is uncoupled from metabolic rate. Our initial assumption is based on literature such as table III in 10.1074/jbc.M408754200 and Fig. 2 in 10.1074/jbc.270.23.13681 (now cited at line 112 along with other literature exploring the connection between intracellular ROS formation and metabolism). This is now explained more clearly at L204-205.

In the equations given in the methods section, what is Gcell?

E1.6 As stated in R1.8 this was an error and has been corrected to wtCell.

In the results section it is not clear how the model progresses, and how one obtains the quantities shown in Fig 1 C and D.

E1.7 Model simulation progresses by solving the coupled set of differential equations (Eq. 1-10) through time, starting with the values given in Table 1, as now explained at the opening of the Methods. (1C is now D and D now E) Fig 1 D is the population size, this can be calculated by multiplying wtCell (molar concentration of GC pairs in the genomes of wt cells) by 6.02e20 (molecules in 1 ml of 1M solution (parameter molML)) and then dividing by 2357528 (the number of GC base-pairs in the *E. coli* MG1655 genome (parameter GCperGen)). This has now been clarified in the figure legend.

E1.8 Fig 1 E is the mutation rate as described in R3.2 and now has an improved description in the figure legend.

This is important for readers to get some intuition for how the model works. Even after looking at the equations I don't know how one gets from all the metabolic stuff to the number of cells, or number of DNA replications. It would really help to have a simple summary of what each equation is doing.

E1.9 We have now included further references in the results section to the equations in the methods section as well as descriptions alongside the equations in the methods section. Additionally, the addition of Appendix 1 adds detail to clarify the ODE modelling process.

I don't know why the base model A generates a positive relationship between cell density and mutation rate – I would expect that there would be no relationship between the two. The explanation provided in the second section of the results is not convincing – are there any data supporting the idea that increased external glucose increases internal glucose, and therefore increases ROS production rate?

E1.10 Figure S3 shows that increased external glucose results in increased internal glucose concentrations (iGlc). As shown in Eq.7, ROS production is linearly related to iGlc by slope ('M1' x 'r'); therefore, higher iGlc leads to increased ROS production which leads to increased mutation rates at higher density. The removal of any reverse DAMP in model B (eGlc and iGlc are uncoupled) and model C (ROS production and iGlc uncoupled) further evidence our conclusions as to the source of reverse DAMP in model A. We have now expanded the explanation given at L193-197.

Could it be that the mutation rate calculation is not accounting for the number of replications (generations)? At higher density (controlled here by higher initial glucose), there are more rounds of cell divisions and therefore more mutant cells.

E1.11 The mutant cells in this model do not divide, therefore mCell measures the number of mutational events having occurred (termed 'm' in the fluctuation assays), as described in E1.3. This simplification makes the direct calculation of mutation rate as mCell/(mCell+wtCell) possible. This is now clarified on L121-123.

I am also concerned that the model does not have a cell death rate,

E1.12 Though cited paper 10.1111/j.1365-2958.2007.05988.x shows the death rate in minimal media to be low we have now explicitly discussed this simplification of models such as ours in L565. This assumption of no death is also in line with the assumptions made in our fluctuation assays which is discussed and challenged on L989-992 & Fig. S18, which now shows that death rates as high as 25% do not substantially (or consistently) alter estimations of DAMP.

and has strange cell states, e.g., with zero internal glucose levels (Fig S2).

E1.13 This is true as the model allows cells to use up all external and internal nutrients and enter stasis in the stationary phase. While the draining of all metabolites is obviously unrealistic, this does not alter the output of the model DAMP slope. This is now illustrated in Fig. A4, Appendix 1 and discussed in the methods on L566-570

The results section reporting analysis of specific mutants to test models D & F is centred on the slope of the relationship between population density and mutation rate. It would be important to also discuss whether the range of mutation rates matches expectation.

E1.14 As the models are fitted separately to give an expected mutation rate (2e-10, Foster et al, 2015) they do not give predictions as to the absolute mutation rate. Instead, their purpose is to give predictions about the relationship between density and mutagenesis. We do however see the overall mutational effects that we would expect in our lab work; for example the anaerobic treatment results in decreased mutations rates, as now discussed in L294 & L476, and the *fur* knockout results in increased mutation rates as one would expect given the increase in free radicals produced via Fenton chemistry as now included in table S1 (column 'MutRatetoRifR').

Reviewer 1 makes a related, and I think important point, about the variable range of cell densities used in the different experiments.

E1.15 We have now performed extra experiments to address this (see R1.2).

Some statements seem to be wrong or are very speculative, and should be either removed or discussed with sufficient support and reasoning. E.g., line 459 ("How DAMP is lost between close evolutionary relatives remains an interesting question and is perhaps linked to the formation of multicellular aggregates by P. aeruginosa"),

E1.16 The question of how DAMP evolves between species has not been addressed and is beyond the scope of this paper, meaning that any statement about it will necessarily be speculative. However, given existing data cited (Krašovec et al, 2017 https://doi.org/10.1371/journal.pbio.2002731) showing that *Pseudomonas aeruginosa* does not show DAMP, it seems worth speculating about what the cause of this may be. We have added to this sentence other potential causes of this difference in DAMP as avenues for future research and have clarified the logic behind these speculations.

line 468 ("in strains with DAMP, the mutation rate decreases as the absolute fitness increases").

E1.17 This is a reiteration of existing published data (Krašovec et al, 2014 https://doi.org/10.1038/ncomms4742) however we have now been more precise rephrasing this as "in wildtype *E. coli* the mutation rate has an inverse relationship not just with population density but also with absolute fitness […](23)".

The sentence beginning on line 393 is odd, because the phrasing implies distinct co-existing species but the experiments here are focused on E coli strains different by a few mutations (the extrapolation itself is fine, and not really surprising; it is just oddly phrased).

E1.18 We have now reworded this sentence to clarify that our data shows cross genotype effects (within species) and that this may lead to hypotheses about cross species effects.

On line 398, the discussion of population structure is puzzling, because the results shown here seem to have no direct bearing on population structure.

E1.19 We have changed the comment on L446-448 to focus on the ecological effects of ROS on ecology rather than population structure as this is more relevant to this study.

Minor point: Fig 1C, y axis label should be population density.

E1.20 Thank you, this has been corrected.
