# Supplementary material for: Collective peroxide detoxification determines microbial mutation rate plasticity in E. coli
Source: PLoS Biol. 2024 Jul 15;22(7):e3002711. doi: 10.1371/journal.pbio.3002711 (PMC11272383; doi:10.1371/journal.pbio.3002711)
Supplement: Attachment — Submitted filename: Responses_R2.docx [file pbio.3002711.s041.docx]

Faculty of Science and Engineering

Michael Smith Building

The University of Manchester

Oxford Road

Manchester

M13 9PL

UK

rok.krasovec@manchester.ac.uk

chris.knight@manchester.ac.uk

+44(0)161 2755378

www.manchester.ac.uk

Responses to comments arising from the first revision of: ‘Collective peroxide detoxification determines mutation rate plasticity in *E. coli*’

12 June 2024

We would like to thank all the reviewers again for their thoughtful and constructive comments which have improved the quality of this revised manuscript. We have included here a response to the second round of comments from reviewers.

Reviewer #1:

It is clear that the authors have made a substantial effort in addressing reviewer comments. The additional results and changes to the manuscript are sufficient to convince me that the the main conclusions of the manuscript are now supported by the data.

On remaining thing that left me confused is the "cumulative mutation rate" used in Fig A3 and Fig 1E. In reply R3.2 the mutation rate in Fig 1E is described as cumulative in a sense that in stationary phase there is no mutation rate so no new mutations are formed and the curve levels out. However in Fig 1E it is not explained that this is cumulative and the figure axis only says Mutation rate. In Fig A3 the axis legend says Cumulative mutation rate but for panel D and F the cumulative mutation rate goes to 0 after 24 h. Please clarify this to help the reader understand what is shown.

We have now updated the figure legend for Fig. 1 to include “E: mutation rate calculated as the ratio of mutated to total base pairs therefore representing the chance of a single base-pair mutating in a single division (generation); this is a cumulative measure of mutation rate in the sense that it considers all the mutations that have accumulated up to the given time, making it directly comparable to experimental measures of mutation rate.” and the caption of Fig. A3 to read “Cumulative mutation rate is defined as all mutant base pairs produced (mCell) divided by all base pairs produced (mCell+wtCell) at each time point. Plots A-K show dynamics for model variants A-K respectively all simulated at initial external glucose concentration of 250mg/L. **Note that although models D and F appear to trend towards 0 both models reach a mutation rate of ~2x10^-10^ after 24 hours (see Fig. 2A).**”.

We believe that these changes make sure readers understand the cumulative nature of the mutation rates. Measuring mutation rates in this way is important for the pairing of simulation and experimental work as the classical Fluctuation Assay method allows only for the estimation of a cumulative mutation rate over the incubation period (in our case 24 hours).

Reviewer #2:

No new comments.

Reviewer #3:
[identifies himself as Christopher J Marx]

I was generally quite positive about the R1 version I saw last, but I am even more convinced by this version. There were many excellent questions raised by the reviewers and editor and I feel like these have been dealt with in a very thorough manner. My concerns, which were mainly those of being able to "see" what the model is actually doing, are assuaged by the figures showing mutation rate as a function of time for all of the different models. I think that is extremely helpful to connect intuition to actual model outputs. I fully support this version of the paper
